# First Balkan Brief Illness Perception Questionnaire (IPQ-B) among high-risk pregnancies

**DOI:** 10.1371/journal.pone.0334844

**Published:** 2025-10-28

**Authors:** Maja Macura, Jovana Todorović, Vedrana Pavlović, Katarina Ivanović, Ivana Novaković, Miloš Milinčić, Miroslava Gojnić

**Affiliations:** 1 Clinic for Gynecology and Obstetrics, University Clinical Centre of SerbiaBelgrade, Serbia; 2 Faculty of Medicine, University of Belgrade, Belgrade, Serbia; 3 Institute of Social Medicine, University of Belgrade, Belgrade, Serbia; 4 Institute for Medical Statistics and Informatics, University of Belgrade, Belgrade, Serbia; Fondazione Policlinico Universitario Agostino Gemelli IRCCS, Universita' Cattolica del Sacro Cuore, ITALY

## Abstract

**Background:**

Pregnancy is a particularly delicate period in which many health-related changes lead to changes in the perception of women’s well-being, in both physiological and especially high-risk pregnancies. In high-risk pregnancies, the relationship between illness perception and general well-being is even more complicated as sometimes there might not be any apparent signs or symptoms, but the pregnant woman or foetus still might be at risk.

**Aims:**

To assess the validity and reliability of the existing Serbian version of the Brief Illness Perception Questionnaire (B-IPQ) in a specific population of pregnant women with high-risk pregnancies (HRP).

**Methods:**

This was a cross-sectional study including 290 patients hospitalized at the Clinic for Gynaecology and Obstetrics, University Clinical Centre of Serbia. The research instrument was a questionnaire with six sections: 1) socio-demographic; 2) pregnancy-related; 3) COVID-19 pandemic–related data 4) B-IPQ; 5) The World Health Organization Quality of Life Brief Version (WHOQOL-BREF) and 6) The Depression, Anxiety and Stress Scale – 21 Items (DASS-21). Psychometric properties of the Serbian version of B-IPQ were analysed through factorial structure and internal consistency (reliability). Confirmatory factor analysis (CFA) was performed to confirm the original two-dimensional structure of the IP.

**Results:**

Analysis of internal consistency of the Serbian version of the eight-item IPQ-B showed that Cronbach’s alpha of the entire scale was 0.7, indicating good scale reliability. IP correlated significantly with QoL related to mental health, stress, anxiety, and depression levels. The consequence domain of IP affected mental health mostly. IP was one of the main direct predictors of QoL and an indirect predictor through depression, anxiety, and stress levels. Marital status, hypertension in pregnancy, fear for health during the COVID-19 pandemic, and being informed during the COVID-19 pandemic had direct negative effects on IP, and indirectly on QoL.

**Conclusions:**

The Serbian version of IPQ-B has good reliability and validity for illness perception in high-risk pregnancies.

## 1. Introduction

Pregnancy is a particularly delicate period in which many health-related changes lead to changes in the perception of women’s well-being in both physiological and especially high-risk pregnancies [1].

There is no universally accepted definition of a high-risk pregnancy. In general, a high-risk pregnancy involves increased health risks for the pregnant woman, fetus or both, meaning that one of them or both are more likely to become ill or die [2].

Illness perception represents the mental representation and personal experience that the patient has about their disease [3]. The perception of the disease is how the patient understands their health condition and its consequences and how they empathize with it [4]. Illness perception is not necessarily negative, and patients can have a proactive attitude and perceive the disease as something they can successfully deal with [5]. The perception of the disease might influence the outcome of the disease [3], and in recent years, a lot of research [6,7] has been focused on examining illness perception in various diseases.

Illness perception is relatively difficult to access during pregnancy. One of the reasons is that pregnancy already represents a state of altered physical and mental health even when it is considered physiological [1]. In high-risk pregnancies, the relationship between illness perception and general well-being is even more complicated as sometimes there might not be any apparent signs or symptoms, but the pregnant woman or fetus still might be at risk [8–11]. Illness perception in healthy pregnant women in the third trimester of pregnancy was proven as a factor that significantly influenced their quality of life [12]. Illness perception was usually assessed using Brief illness perception questionnaire [13–15], in other recent studies, self-designed questionnaire [16] or illness perception visual analogue scale [17] was used.

As many of the life-threatening conditions in pregnancy have no signs and symptoms [9,11], the illness perception among these patients, may be important for disease management and treatment compliance. Because pregnancy is such a delicate state, a pregnant patient is not only worried about her own well-being but also about her offspring [18] making this a unique disease perception. To the best of our knowledge, although the studies that used a Brief illness perception questionnaire were previously conducted in Serbia [19], there were no studies that examined illness perception in pregnant women with high-risk pregnancies. This research gap is evident not only in Serbia and other Balkan countries but also on a global scale, with limited studies available on the subject published in last few years [20,21]. The aim of this study was to assess the validity and reliability of the existing Serbian version of the Brief Illness Perception Questionnaire (IPQ-B) in a specific clinical context, i.e., population — pregnant women with high-risk pregnancies. While IPQ-B has been previously used in Serbian-speaking populations, to our knowledge, this is the first study to formally validate its psychometric properties in a sample of hospitalised high-risk pregnant women.

In addition, the purpose of the path analysis was to determine the factors contributing to the quality of life in pregnant patients hospitalized for high-risk pregnancy management. We hypothesized that increasing levels of depression, stress, and anxiety negatively affect quality of life by maladaptive illness perception.

## 2. Materials and methods

The cross-sectional study included 290 patients hospitalized at the Department of Pathological Pregnancies in the Clinic for Gynecology and Obstetrics at the University Clinical Center of Serbia (CGO UCCS). The study was conducted in October 2022-April 2023. Patients were asked to fill in the anonymous questionnaire during their hospitalization in CGO UCCS. There was no age related exclusion criteria.

The patients were given oral information about the study and were then asked to fill in an anonymous questionnaire. It was considered that all patients who had filled in and returned the questionnaires gave their consent for participation in the research. The study was approved by the Ethics Committee of the Faculty of Medicine of the University of Belgrade.

The decision to include perceptions of the novel coronavirus disease (2019-nCoV) in this study was driven by its relevance to both health and social factors affecting pregnant women. Beyond the direct implications of vaccination and health outcomes, the pandemic introduced significant social challenges, such as restrictions on hospital visits and the absence of partners during delivery due to strict epidemiological measures. These circumstances highlighted the importance of examining the perception of COVID-19 to fully capture its impact on the experiences of pregnant women.

### 2.1. Questionnaires

The research instrument consisted of six sections:1)socio-demographic and -economic data such as: age, marital status, education level, and employment status; 2) pregnancy-related data such as: current body weight, body height, gestational age, conception method, and reproductive history (previous live births, miscarriages and abortions) why they are hospitalised and if they have any other comorbidities; 3) Perception of COVID-19 pandemic and attitudes towards vaccination during pregnancy. 4) Brief Illness perception questionnaire (IPQ-B) [4], 5) The World Health Organization Quality of Life Brief Version (WHOQOL-BREF) [22] and 6) The Depression, Anxiety and Stress Scale – 21 Items (DASS-21) [23].

IPQ-B consists of nine questions related to a certain aspect of the perception of the disease, such as: consequences of the disease, assessment of the time course of the disease, personal control, treatment control, intensity, illness concern, disease understanding, emotional response and disease cause perception. The answers are provided on a scale ranging from 0 to 10, and the last question is an open-ended question where the patient can fill out their opinion of the causes of their pathological pregnancy [24]. Since validation of the IPQ-B questionnaire in the population of high-risk pregnancies was one of the aims of this study, the questionnaire was adapted to specifically target high-risk pregnancy as a disease. The IPQ-B was translated to the Serbian language and back, according to the WHO recommendations [25].

The psychometric properties of the IPQ – B as detailed in the systematic review [4] include: Concurrent validity, predictive validity, discriminant validity, sensitivity to change, reliability, flexibility and cross-cultural validation. These properties indicate that the IPQ– B is a valid and versatile tool for assessing illness perceptions in diverse populations and settings.

The WHOQOL-BREF is a questionnaire on participant’s well-being in the previous two weeks. Answers are represented on a 1–5 Likert scale. Questions are organized into 4 domains: physical health (6 questions), psychological health (6 questions), social relationships (3 questions), and environment (8 questions) [22].

The DASS-21 is a questionnaire created to quantify levels of depression, anxiety and stress in the previous week. Answers are represented on a 1–4 Likert scale. Questions are organized into 3 domains (depression, anxiety and stress) each containing 7 questions [23].

### 2.2. Statistical analysis

Descriptive statistics, including means and confidence intervals for numerical variables, and numbers and percentages for categorical variables were used to characterize the study sample. The sample size estimation was based on the assumption needed to be fulfilled for the application of factor analysis, set by Tabachnick and Fidell [26], where the minimum number of respondents must be 150, with at least 5 respondents for each item. Psychometric properties of the Serbian version of the IPQ were analyzed through the analysis of factorial structure and internal consistency (reliability). The internal consistency of the IP was assessed by using Cronbach’s alpha coefficient (ranges from 0–1, the latter meaning perfect reliability) and McDonald’s omega coefficient. Confirmatory factor analysis (CFA) was performed to confirm the original two-dimensional structure of the IP. The absolute goodness-of-fit of the two-dimensional model was evaluated using the chi-square test (values that are < 0.05 signify that a model may be a bad fit for the data, whereas χ2 values > 0.05 may render the model a good fit) and three additional fit measures - Comparative-Fit Index (CFI), Incremental Fit Index (IFI) and the Root Mean Square Error of Approximation (RMSEA). Values of CFI and IFI above 0.95 are considered adequate, whereas RMSEA values below 0.06 indicate an acceptable model fit. To complement the classical test theory approach, an Item Response Theory (IRT) analysis was conducted using the graded response model (GRM) implemented in the mirt package [27] for R [28]. Model evaluation included item parameters, S-X^2^ item-fit statistics, and test information and reliability functions. Path analysis was used as it allowed the assessment of the direct and indirect effects of the predictors through simultaneous modelling of related regression relationships. Before assessing the direct and indirect paths among the variables, the absence of multicollinearity was verified using Pearson’s correlation coefficient (r), tolerance, and variance inflation factor (VIF). The estimates were within acceptable ranges: r < 0.8, tolerance ≥ 0.1, and VIF ≤ 10. For continuous variables, the skewness and kurtosis coefficients were below 1. Multiple measures were used to determine the adequacy of model-fit to the data; these included the following fit indices: the χ 2 test, the comparative fit index (CFI), the Tucker–Lewis index (TLI), the normed fit index (NFI), and the root mean square error of approximation (RMSEA). All fit indices were used to indicate the degree to which a pattern of fixed and free parameters specified in the model was consistent with the pattern of variances and covariances from a set of observed data. If the p-value resulting from a χ 2 test is greater than 0.05, the path model is considered to have a good fit. A value of the χ 2 test less than two times its degree of freedom is considered favourable. For the RMSEA, if the value is less than 0.05, the model is considered to have a good fit. If the CFI, TLI, and NFI values are greater than 0.95, the model is considered to have a good fit. In the model, the arrows demonstrate the direction of the hypothesized association. Standardized regression coefficients are presented as path estimates, demonstrating the strength of the path between variables. To enable comparison between variables, the standardized effects were estimated to show path coefficients on a common scale ranging from −1–1. After controlling for other predictors in the model, the direct coefficient shows the effect of an independent variable on a dependent variable, whereas the indirect coefficient shows the effect of an independent variable on a dependent variable which is mediated by variables on the path. The sum of the direct and indirect effects is the total effect, connecting the two variables. In all analyses, the significance level was set at 0.05. Confirmatory factor analysis (CFA) and path analysis were done using Amos 21 (IBM SPSS Inc., Chicago, IL, USA, 2012). The entire sample was used. All other statistical analysis were performed using IBM SPSS Statistics 25 software.

## 3. Results

A total of 290 women with high-risk pregnancies completed the Serbian version of the IPQ. The mean age of study participants was 31.4, with a range of 16–49, while the mean gestational age was 30.0 weeks with a range of 8–41. Most participants (89.3%) had a partner. The majority of the participants had secondary education or below (53.8%). More than half of participants (72.8%) were employed. Diabetes mellitus was present in 37.9% of women, thrombophilia in 15.2%, and hypertension in pregnancy was present in 14.8% of women. Detailed demographic and clinical characteristics of the study population are presented in Table 1.

**Table 1:** Sociodemographic and health-related status of participants.

Variables	n = 290
Age, mean (95% CI)	31.4 (30.7-32.1)
BMI, mean (95% CI)	27.9 (27.2-28.6)
Week of gestation, mean (95% CI)	30.0 (29.2-30.9)
Marital status, n (%)	
Single	30 (10.4)
Married/ Domestic partnership	259 (89.3)
Level of education, n (%)	
Primary education and below/Secondary education	156 (53.8)
Tertiary education or above	134 (46.2)
Employed, n (%)	211 (72.8)
Conception, n (%)	
Spontaneously	266 (92.4)
Artificial fertilization	22 (7.6)
Threatened preterm labour, n (%)	72 (24.9)
Hypertension in pregnancy, n (%)	43(14.8)
Diabetes mellitus, n (%)	110 (37.9)
Thrombophilia, n (%)	44 (15.2)
Pregnancy after Cesarean section, n (%)	25 (8.6)
First pregnancy, n (%)	111 (38.3)
Parity, n (%)	146 (50.3)
Miscarriages, n (%)	36 (12.4)
DASS-21, mean (95% CI)	
Stress	3.55 (3.1-4.0)
Anxiety	4.4 (3.9-4.9)
Depression	4.0 (3.6-4.4)
WHOQoL, mean (95% CI)	
Physical health	15.9 (15.6-16.2)
Mental health	16.1 (15.8-16.4)
Social relationships	16.9 (16.5-17.2)
Environment	15.7 (15.4-16.1)

Mean DASS-21 stress, anxiety, and depression scores were 3.55 (95% CI 3.10–4.00), 4.39 (95% CI 3.91–4.86), and 3.99 (95% CI 3.56–4.42), respectively. Mean WHOQOL -BREF subscales scores for physical health, mental health, social relationships, and environmental health were 15.92 (95% CI 15.59–16.24), 16.13 (95% CI 15.83–16.42), 16.86 (95% CI 16.50–17.22), and 15.74 (95% CI 15.43–16.06), respectively.

To the question of whether they feel more fear for their health now compared to the period before the COVID-19 pandemic, 30% of women answered “yes”. A high number of respondents felt that during the pandemic they were sufficiently informed by the relevant sources about the consequences of the COVID-19 infection on pregnancy and breastfeeding (70.2%).

Descriptive statistics of the various IPQ-B dimensions are shown in Table 2. The highest mean score was 3.83 (95% CI 3.43–4.24) for illness concern, while the lowest mean score was 2.44 (95% CI 2.10–2.78) for personal control. Analysis of the internal consistency of the Serbian version of the eight-item IPQ-B showed that Cronbach’s alpha of the entire scale was 0.7 and McDonald’s omega was 0.85, both indicating good scale reliability.

**Table 2:** Average scores of the IPQ-B according to items and domains.

Item	Min	Max	Mean	95% CI	Cronbach’s Alpha If item deleted
***Cognitive illness***					
Consequences	0	10	2.65	2.3-3.0	0.578
Timeline	0	10	3.36	2.9-3.8	0.659
Personal control	0	10	2.44	2.1-2.78	0.599
Treatment control	0	10	2.96	2.5-3.4	0.750
Identity	0	10	2.56	2.2-2.9	0.595
***Emotional***					
Illness concern	0	10	3.83	3.4-4.2	0.620
Emotional	0	10	3.23	2.8-3.7	0.750
Coherency	0	10	2.79	2.4-3.2	0.591
**Total IP**	0	74	23.81	22.1-25.6	0.680

**Fig 1:**
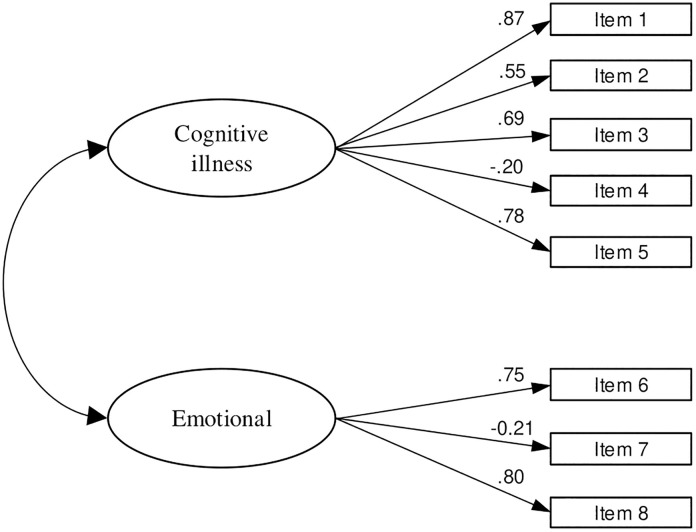
Standardized factor loadings for the IPQ-B questionnaire in Serbian language (eight-item model).

First, the CFA model was conducted on the entire sample to test the eight-item two-factor model fit. Due to the large sample size, the Chi-square test rejected the two-dimensional model (χ2 = 203.156 p < 0.001). The fit indices NFI (0.794), IFI (0.810), and CFI (0.808), along with the RMSEA value of 0.183 (90% CI: 0.161–0.206) did not meet the recommended thresholds for adequate model fit. All standardized factor loadings were statistically significant, ranging from 0.20 to 0.87 (Fig 1).

**Fig 2:**
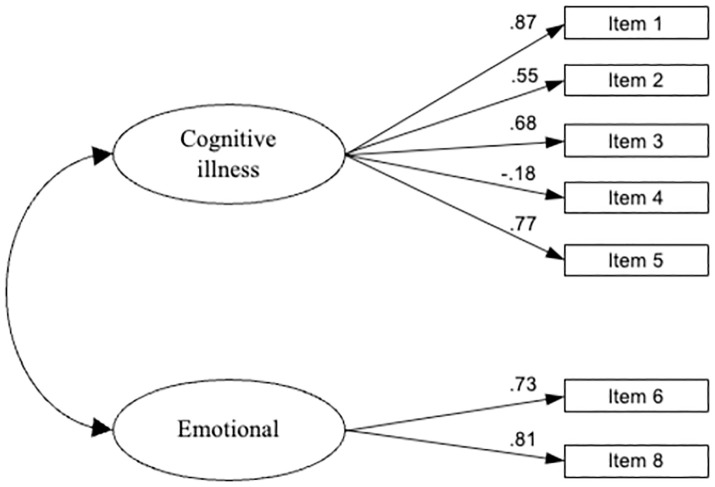
Standardized factor loadings for the IPQ-B questionnaire in Serbian language (seven-item model).

Next, the CFA model was performed on the same sample to test the seven-item (with the emotional item 7 dropped), two-factor (cognitive and emotional) model fit. The seven-item two-factor structure of the IP demonstrated a good fit of the data to the hypothesized model. The Chi-square test rejected the two-dimensional model (χ^2^ = 23.834, p = 0.008), as we expected, due to the large sample size. In contrast, values for fit indices NFI (0.971), IFI (0.983) and CFI (0.983) were above the cut-off of ≥0.95. The RMSEA value of 0.069 (90% CI: 0.033–0.105) was below the suggested value of ≤0.08. All standardized factor loadings were statistically significant and ranged from 0.18 to 0.87 (Fig 2).

The GRM showed strong item discrimination for Item 1 (*a* = 4.31), Item 3 (*a* = 2.29), Item 5 (*a* = 2.70), Item 6 (*a* = 2.25), and Item 8 (*a* = 2.46), moderate for Item 2 (*a* = 1.48), and weak for Item 4 (*a*=−0.52). Thresholds were ordered for all items except Item 4. Limited-information item-fit indices (S-X²) indicated adequate fit for most items, although Items 2, 4, and 6 showed misfit (p < 0.05). The test information curve demonstrated excellent precision in the mid-range of illness perceptions (peak at θ = 0.50, information = 17.23, conditional reliability 0.94), with reliable measurement across θ values from −1.5 to +2.0. Overall, the IRT findings support the reliability of the 7-item B-IPQ and suggest that the scale provides reliable measurement in the average-to-above-average range of illness perception.

The total IPQ-B score correlated significantly with the WHOQoL-BREF mental health subscale score (r = −0.402; p < 0.001) indicating adequate validity between the two instruments. All items of IPQ-B correlated significantly with the WHOQoL-BREF mental health subscale score, except perception of illness duration (Item 2). DASS-21 stress, anxiety, and depression subscale scores correlated significantly with the total IPQ-B scale (r = 0.492; p < 0.001, r = 0.441; p < 0.001 and r = 0.434; p < 0.001, respectively) (Table 3).

**Table 3:** Correlations between IPQ-B items and WHO QoL -BREF mental health subscale score, the DASS-21 stress, anxiety, and depression scores.

Item	QoL Mental health	DASS-21 Stress	DASS-21 Anxiety	DASS-21 Depression
***Cognitive illness***				
Consequences	−0.342*	0.375*	0.314*	0.318*
Timeline	−0.113	0.156*	0.100	0.112
Personal control	−0.225*	0.296*	0.247*	0.246*
Treatment control	−0.190*	0.171*	0.171*	0.182*
Identity	−0.273*	0.380*	0.297*	0.312*
***Emotional***				
Illness concern	−0.184*	0.315*	0.272*	0.289*
Emotional	−0.184*	0.136*	0.162*	0.120*
Coherency	−0.332*	0.437*	0.465*	0.420*
**Total IPQ-B**	−0.402*	0.492*	0.441*	0.434*

*statistically significant correlations.

**Fig 3:**
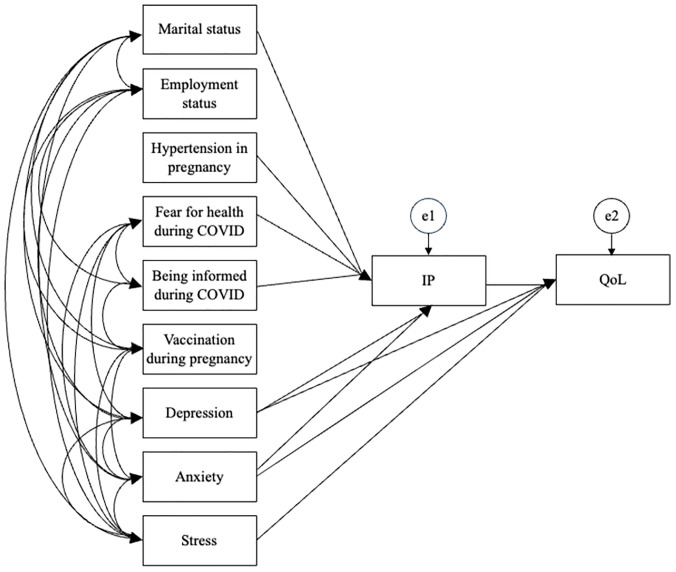
Path model (IP) illness perception. (QoL) quality of life.

The hypothesized relationships among the predicting variables were tested by path analysis, using a maximum likelihood estimate (Fig 3). Standardized coefficient (B) was used to estimate the predicting effects. The best fit of the path model was achieved with χ2 = 36.105, df = 26, CMIN/DF = 1.389, p = 0.090, NFI = 0.940, TLI = 0.961, IFI = 0.983, CFI = 0.982, and RMSEA = 0.037. The constructed path model accounted for 25.5% of the illness perceptions, and 39.1% of the quality of life.

According to this model, illness perception, depression, anxiety and stress were the main direct predictors of the quality of life. Among variables that directly affected the QoL, stress had the highest effect (B = −0.29) and depression (B = −0.17) had the lowest effect. The significant mediating role of illness perception was also identified in the model. Marital status, hypertension in pregnancy, depression, anxiety, fear for health during COVID-19, and being informed during COVID-19 had significant indirect effects on quality of life via illness perception. Being single, the presence of hypertension during pregnancy, depressive and anxiety symptoms, greater fear for health during COVID-19, and being uninformed during COVID-19 are associated with more negative illness perceptions in pregnant women. Depression and anxiety variables from both direct and indirect paths through illness perception had an impact on QoL (Table 4, Fig 3).

**Table 4:** Direct, indirect, and total effects of multiple determinants on Quality of life.

	B	SE	*p*
**Direct**			
IP → QoL	−0.173	0.059	0.014
Depression→QoL	−0.170	0.069	0.010
Anxiety→QoL	−0.194	0.057	0.010
Stress→QoL	−0.292	0.062	0.010
Marital status→IP	−0.157	0.055	0.010
Hypertension in pregnancy→IP	0.128	0.060	0.030
Depression→IP	0.184	0.054	0.010
Anxiety→IP	0.175	0.057	0.010
Fear for health during COVID-19	0.200	0.057	0.010
Being informed during COVID-19	−0.181	0.056	0.010
**Indirect**			
Marital status→IP → QoL	0.027	0.015	0.019
Hypertension in pregnancy→ IP → QoL	−0.022	0.013	0.004
Depression→ IP → QoL	−0.032	0.017	0.019
Anxiety→ IP → QoL	−0.030	0.014	0.017
Fear for health during COVID-19 IP → QoL	−0.035	0.015	0.015
Being informed during COVID→ IP → QoL	0.031	0.013	0.014
**Total**			
IP → QoL	−0.173	0.059	0.014
Marital status→ QoL	0.027	0.015	0.019
Hypertension in pregnancy→ QoL	−0.022	0.013	0.004
Depression→ QoL	−0.202	0.066	0.010
Anxiety→ QoL	−0.225	0.052	0.010
Stress→ QoL	−0.292	0.062	0.010
Fear for health during COVID→ QoL	−0.035	0.015	0.015
Being informed during COVID→ QoL	0.031	0.013	0.014

## 4. Discussion

This study examined the validity and reliability of the Serbian version of B-IPQ in the population of women with high-risk pregnancies. The study showed that the Serbian version of this questionnaire had good reliability and validity, and could be used for the illness perception in high-risk pregnancy.

The population consisted of 290 patients hospitalized for high-risk pregnancy surveillance and delivery planning.

The primary limitation of this study was that the sample included only hospitalized patients. We limited our sample to hospitalized patients to ensure a controlled environment for data collection and to focus on cases where the conditions under investigation were closely monitored. This approach was chosen to minimize variability in patient management and ensure the reliability of our findings in this initial phase of research. As this represents preliminary research on the topic, future studies should be prospective and aim to include high-risk pregnancies managed in outpatient settings to provide a more comprehensive understanding. Moreover, another limitation of our study was a cross-sectional design that was necessary for questionnaire validation.

Most pregnancies were spontaneous and the main cause for hospitalization was threatened preterm labour, diabetes in pregnancy, thrombophilia and hypertension in pregnancy. These results are similar to those published by French and Brazilian research groups that reported that the most frequent admission diagnoses were false and threatened preterm labour, diabetes, infection and hypertensive disease of pregnancy [29,30].

Our results suggest that illness perception correlated significantly with quality of life related to mental health (except perception of illness duration) as well as stress, anxiety, and depression levels. The consequence domain of illness perception affected mental health mostly. This can probably be explained by the future mother’s worry for the baby. A recently published German study [31] evaluated mental health in pregnancies with gestational diabetes Mellitus (GDM) and found that illness perception affects patients who experienced depressive symptoms even though they haven’t used the same questionnaires as research tools. Unlike our study, their focus was only on patients with GDM who were treated as outpatients. In contrast, an Australian study [32], used the same tool as in our research, i.e., IPQ-B, and had the same conclusions as ours, as well as Rieß et al., demonstrating that the concern for adverse effects on baby’s health was one of the most important factors of poor illness perception and consequently QoL. In addition, Maugire’s research group discovered the same effect proven in our study, i.e., that more intense depressive and anxiety symptoms are correlated with more negative GDM illness perceptions.

Our model demonstrated that illness perception was one of the main direct predictors of quality of life, as well as an indirect predictor through depression, anxiety and stress levels. Marital status, hypertension in pregnancy, fear for health during covid pandemic, and being informed during covid pandemic had direct negative effects on illness perceptions, and therefore on QoL indirectly.

Analysis of the internal consistency of the Serbian version of the eight-item IPQ-B showed that Cronbach’s alpha of the entire scale was 0.7, indicating good scale reliability. Similar results were published for the Norwegian version of IPQ-B in terms of good reliability and validity in sub-acute and chronic low back pain [33], as well as for the Portuguese version of IPQ-B in periodontal disease [34] with Cronbach’s alpha of 0.7 and 0.8 respectively.

Our initial eight-item model did not show a good fit in the CFA, indicating less than ideal construct validity. The second conducted CFA with the one eliminated item (item No. 7) showed a good model fit. The seven-item two-factor model, therefore, showed relatively good construct validity in our study. Similar results were obtained by study groups that analyzed IPQ-B in Turkish cancer and periodontal disease patients [35,36], as well as Indonesians with Type 2 Diabetes Mellitus [37].

Maguire et al. [32] demonstrated that depression and anxiety had a significant effect on maladaptive illness perception, which is in line with our findings, but the benefit of our study comes from the fact that we observed various pathologies that made pregnancy qualify as high-risk. A recent study by Scime et al.[38] showed that worse illness perception in pregnant women with chronic disease decreased their motivation for breastfeeding, which is a solid example of how illness perception can have more profound personal and public health consequences.

Marital status and depression levels were also factors that had a negative impact on QoL in pregnant patients in a recent Greek study by Saridi et al [39]. On the other hand, our research doesn’t support the fact that the third trimester negatively impacts the quality of life, however, 83% of Saradi et al’s sample had an uncomplicated pregnancy.

Another recent study [40] showed that women in the third trimester and nulliparous women had the worst health related quality of life. Again, this study didn’t include unmarried women or women with chronic diseases. Similarly, in a cross-sectional study from Pakistan, pregnancy trimester, education, marital status and income were identified as factors that affect the quality of life in physiological pregnancy [41].

Overall, this discrepancy supports the fact that women with high-risk pregnancies have diminished quality of life due to maladapted illness perception rather than regular pregnancy changes of life such as a more sedentary and isolated lifestyle in late pregnancy.

## 5. Conclusion

In conclusion, the Brief Illness Perception Questionnaire (IPQ-B) is a good tool for fast assessment of illness perception in high-risk pregnancies, and it demonstrates good reliability and validity. Our model demonstrated that illness perception was one of the main direct predictors of quality of life, as well as an indirect predictor through depression, anxiety and stress levels.

In the future, we plan to conduct a prospective research expanding it to a more diverse population of high-risk pregnancies, including patients managed in outpatient settings.

Moreover, implementing this brief and time-efficient questionnaire in patients with high-risk pregnancies could help identify those with maladaptive illness perceptions, prompting clinicians to provide appropriate reassurance and interventions to improve their quality of life. Additionally, it may detect patients who are reluctant to discuss depression or anxiety due to stigma but are more comfortable addressing pregnancy-related concerns, enabling obstetricians to refer them to mental health professionals when necessary.

## Supporting information

S1 File Ethics Committee approval in the original form. (JPG)

S2 File Ethics Committee approval in English translation form. (DOCX)

S3 File Author changes declaration. (PDF)

S4 File IPQ-B in the original form. (PDF)

S5 File IPQ-B in Serbian translation form. (PDF)

S6 File STROBE checklist. (DOC)

S7 File CFA All questions. (AMOSOUTPUT)

S8 File CFA. (AMOSOUTPUT)

S9 File Database. (SAV)

S10 File Output. (SPV)

S11 File PathAmosOutput. (AMOSOUTPUT)
