# Supplementary figures and images for: First Balkan Brief Illness Perception Questionnaire (IPQ-B) among high-risk pregnancies
Source: PLoS One. 2025 Oct 28;20(10):e0334844. doi: 10.1371/journal.pone.0334844 (PMC12561911; doi:10.1371/journal.pone.0334844)

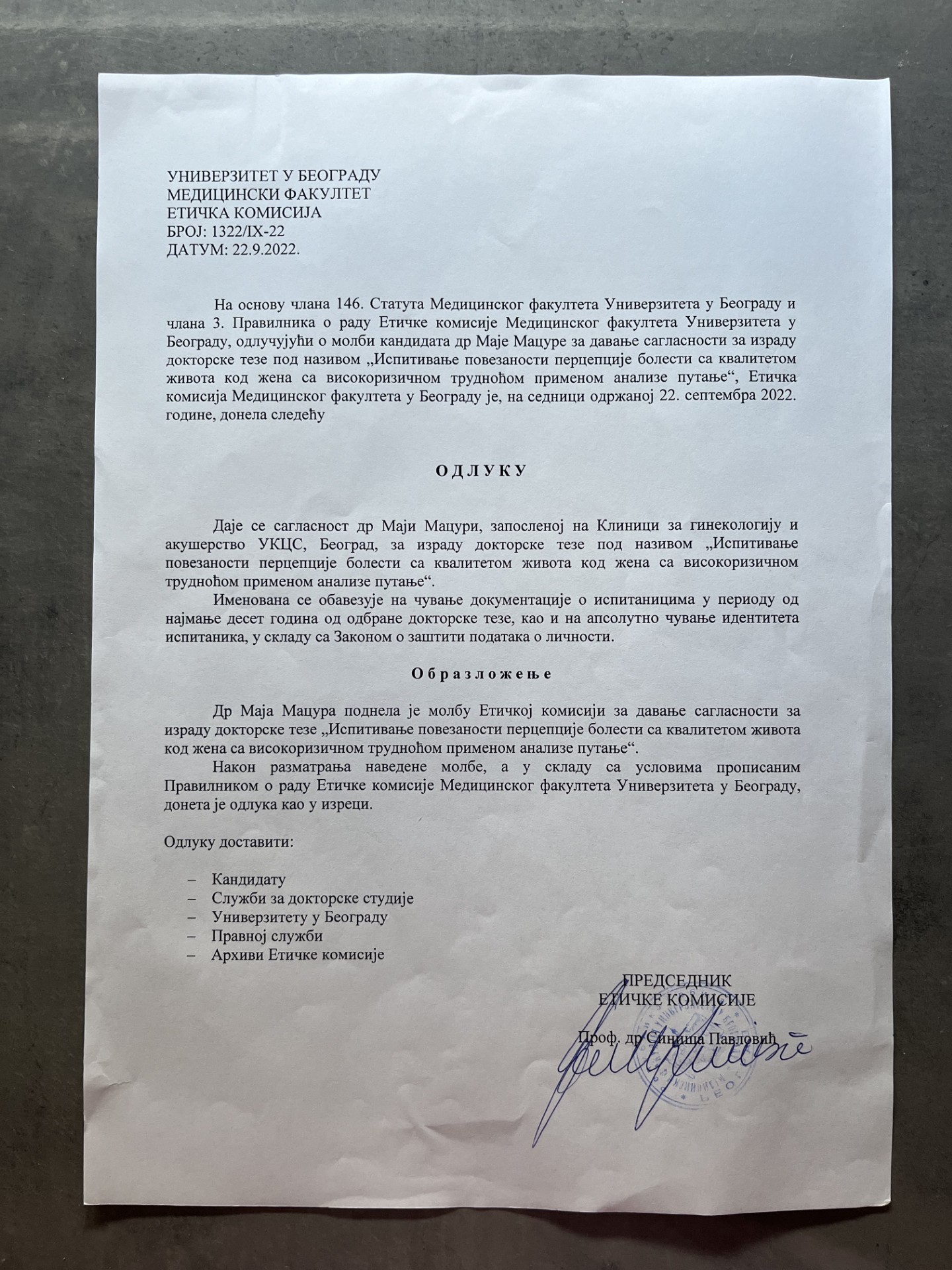

Supplement: S1 File — (JPG) [file pone.0334844.s001.jpg]
