# Supplementary material for: First Balkan Brief Illness Perception Questionnaire (IPQ-B) among high-risk pregnancies
Source: PLoS One. 2025 Oct 28;20(10):e0334844. doi: 10.1371/journal.pone.0334844 (PMC12561911; doi:10.1371/journal.pone.0334844)
Supplement: S2 File — (DOCX) [file pone.0334844.s002.docx]

UNIVERSITY OF BELGRADE
FACULTY OF MEDICINE
ETHICS COMMISSION
NUMBER: 1322/IX-22
DATE: 22.9.2022.

Based on Article 146. of the Statute of the Faculty of Medicine of the University of Belgrade and Article 3. of the Rules of Procedure of the Ethics Commission of the Faculty of Medicine of the University of
Belgrade, deciding on the request of candidate Maja Macura, MD., for granting approval for the research for doctoral theses research titled "Examination of the relationship between illness perception and quality of life in women with high-risk pregnancies using path analysis", Ethics commission of the Faculty of Medicine in Belgrade, at the session held on September 22, 2022, brought the following

THE DECISION

Consent is given to Maja Macura, MD, an employee at the Clinic for Gynecology and obstetrics, University Clinical Center of Serbia, Belgrade, for the research for doctoral theses titled " Examination of the relationship between illness perception and quality of life in women with high-risk pregnancies using path analysis ".
The appointee is obliged to keep the documentation of the respondents in the period from
at least ten years after the approval of the doctoral thesis, as well as the absolute preservation of the identity of the respondents, in accordance with the Personal Data Protection Act.

THE RATIONALE

Maja Macura, MD, submitted an application to the Ethics Commission for approval for
doctoral thesis " Examination of the relationship between illness perception and quality of life in women with high-risk pregnancies using path analysis ".
After considering the mentioned request, and in accordance with the prescribed conditions
By the Rulebook on the work of the Ethics Commission of the Faculty of Medicine of the University of Belgrade, a decision was made as in the saying.

Submit the decision:
- To the candidate
- Services for doctoral studies
- University in Belgrade
- Legal services
- Archives of the Ethics Commission

President of the Ethics Commission

Prof. dr Siniša Pavlović
