# Supplementary material for: First Balkan Brief Illness Perception Questionnaire (IPQ-B) among high-risk pregnancies
Source: PLoS One. 2025 Oct 28;20(10):e0334844. doi: 10.1371/journal.pone.0334844 (PMC12561911; doi:10.1371/journal.pone.0334844)
Supplement: S3 File — (PDF) [file pone.0334844.s003.pdf]

SUPPLEMENTARY MATERIAL: AUTHOR CHANGES

In the original paper writing, the author Ivanovic K. worked under the supervision of the previous author Dugalic S. After the authors had decided to resubmit the paper, Ivanovic K. independently reviewed and participated in the draft reconstruction which is why Dugalic S. as her previous supervisor, has decided that she claimed a greater right to be the author. All the authors have agreed upon this decision including Dugalic S.
