# Supplementary material for: First Balkan Brief Illness Perception Questionnaire (IPQ-B) among high-risk pregnancies
Source: PLoS One. 2025 Oct 28;20(10):e0334844. doi: 10.1371/journal.pone.0334844 (PMC12561911; doi:10.1371/journal.pone.0334844)
Supplement: S5 File — (PDF) [file pone.0334844.s005.pdf]

Za naredna pitanja zaokružite broj koji najbolje opisuje vaše stanje:

1. U kojoj meri Vaša bolest utiče na Vaš život?

0 1 2 3 4 5 6 7 8 9 10

Ne utiče uopšte	Znatno utiče

2. Do kad smatrate da će bolest trajati?

0 1 2 3 4 5 6 7 8 9 10

Veoma kratko	Zauvek

3. U kojoj meri imate osećaj da kontrolišete svoju bolest?

0 1 2 3 4 5 6 7 8 9 10

Apsolutno kontrolišem	Ekstremno bez kontrole

4. U kojoj meri mislite da Vam terapije i tretmani pomažu u lečenju Vaše bolesti?

0 1 2 3 4 5 6 7 8 9 10

Ne pomažu uopšte	Izuzetno pomažu

5. U kojoj meri osećate simptome bolesti u trudnoći?

0 1 2 3 4 5 6 7 8 9 10

Nemam<br>simptome	Izrazito

6. U kojoj meri ste zabrinuti svojom bolešću?

0	1	2	3	4	5	6	7	8	9	10

Nisam uopšte<br>zabrinuta	Izrazito<br>zabrinuta

7. U kojoj meri mislite da razumete svoju bolest?

0	1	2	3	4	5	6	7	8	9	10

Ne razumem<br>uopšte	Razumem<br>veoma jasno

8. U kojoj meri Vas bolest remeti emotivno? (da li ste ljuti, uplašeni, uznemireni, depresivni)

0	1	2	3	4	5	6	7	8	9	10

Ne remeti me<br>emotivno	Znatno<br>me remeti

9. Navedite i poređajte po važnosti tri najbitnija uzroka koja su doprinela pojavi Vaše bolesti:

1.______________________________

2.______________________________

3.______________________________
