# Supplementary material for: First Balkan Brief Illness Perception Questionnaire (IPQ-B) among high-risk pregnancies
Source: PLoS One. 2025 Oct 28;20(10):e0334844. doi: 10.1371/journal.pone.0334844 (PMC12561911; doi:10.1371/journal.pone.0334844)
Supplement: S6 File — (DOC) [file pone.0334844.s006.doc]

STROBE Statement—Checklist of items that should be included in reports of ***cross-sectional studies***

|  | Item No | Recommendation |
| --- | --- | --- |
| **Title and abstract** | 1 | (*a*) Indicate the study’s design with a commonly used term in the title or the abstract  ✓This was a cross-sectional study including 290 patients hospitalized at the Clinic for Gynaecology and Obstetrics, University Clinical Centre of Serbia. |
|  |  | (*b*) Provide in the abstract an informative and balanced summary of what was done and what was found  ✓Analysis of internal consistency of the Serbian version of the eight-item IPQ-B showed that Cronbach’s alpha of the entire scale was 0.7, indicating good scale reliability. IP correlated significantly with QoL related to mental health, stress, anxiety, and depression levels. The consequence domain of IP affected mental health mostly. IP was one of the main direct predictors of QoL and an indirect predictor through depression, anxiety, and stress levels. Marital status, hypertension in pregnancy, fear for health during the COVID-19 pandemic, and being informed during the COVID-19 pandemic had direct negative effects on IP, and indirectly on QoL. |
| Introduction | | |
| Background/rationale | 2 | Explain the scientific background and rationale for the investigation being reported  ✓ Because pregnancy is such a delicate state, a pregnant patient is not only worried about her own well-being but also about her offspring (18) making this a unique disease perception. To the best of our knowledge, although the studies that used a Brief illness perception questionnaire were previously conducted in Serbia (19), there were no studies that examined illness perception in pregnant women with high-risk pregnancies. |
| Objectives | 3 | State specific objectives, including any prespecified hypotheses  ✓The aim of this study was to assess the validity and reliability of the existing Serbian version of the Brief Illness Perception Questionnaire (IPQ-B) in a specific clinical context i.e. population — pregnant women with high-risk pregnancies. While IPQ-B has been previously used in Serbian-speaking populations, to our knowledge, this is the first study to formally validate its psychometric properties in a sample of hospitalised high-risk pregnant women. In addition, the purpose of the path analysis was to determine the factors contributing to the quality of life in pregnant patients hospitalized for high-risk pregnancy management. |
| Methods | | |
| Study design | 4 | Present key elements of study design early in the paper  ✓The cross-sectional study included 290 patients hospitalized at the Department of Pathological Pregnancies in the Clinic for Gynecology and Obstetrics at the University Clinical Center of Serbia (CGO UCCS). The study was conducted in October 2022-April 2023. Patients were asked to fill in the anonymous questionnaire during their hospitalization in CGO UCCS. The patients were given oral information about the study and were then asked to fill in an anonymous questionnaire. It was considered that all patients who had filled in and returned the questionnaires gave their consent for participation in the research. |
| Setting | 5 | Describe the setting, locations, and relevant dates, including periods of recruitment, exposure, follow-up, and data collection  ✓The cross-sectional study included 290 patients hospitalized at the Department of Pathological Pregnancies in the Clinic for Gynecology and Obstetrics at the University Clinical Center of Serbia (CGO UCCS). The study was conducted in October 2022-April 2023. The research instrument consisted of six sections:1)socio-demographic and -economic data such as: age, marital status, education level, and employment status; 2) pregnancy-related data such as: current body weight, body height, gestational age, conception method, and reproductive history (previous live births, miscarriages and abortions) why they are hospitalised and if they have any other comorbidities; 3) Perception of COVID-19 pandemic and attitudes towards vaccination during pregnancy. 4) Brief Illness perception questionnaire (IPQ-B) (4), 5) The World Health Organization Quality of Life Brief Version (WHOQOL-BREF)(22) and 6) The Depression, Anxiety and Stress Scale - 21 Items (DASS-21) (23) |
| Participants | 6 | (*a*) Give the eligibility criteria, and the sources and methods of selection of participants  ✓The patients were given oral information about the study and were then asked to fill in an anonymous questionnaire. It was considered that all patients who had filled in and returned the questionnaires gave their consent for participation in the research. There was no age related exclusion criteria. |
| Variables | 7 | Clearly define all outcomes, exposures, predictors, potential confounders, and effect modifiers. Give diagnostic criteria, if applicable  ✓The main study outcome was the level of illness perception, assessed using Serbian version of the Brief Illness Perception Questionnaire (B-IPQ). Explanatory variables included sociodemographic characteristics (age, marital status, education level, and employment status), pregnancy-related variables (gestational age, conception method, reproductive history, BMI and comorbidities), COVID-19–related variables ( perceived fear for health during the pandemic and adequacy of information received, psychological and quality of life measures (Depression, Anxiety and Stress Scale – 21 items (DASS-21) and the WHOQOL-BREF). Diagnostic criteria for comorbidities (e.g., diabetes, hypertension, thrombophilia) were obtained from patient medical records at the time of hospitalization. |
| Data sources/ measurement | 8* | For each variable of interest, give sources of data and details of methods of assessment (measurement). Describe comparability of assessment methods if there is more than one group  ✓All data were collected using a structured questionnaire administered to pregnant women during hospitalization in a high-risk pregnancy unit. Illness perception was measured with the Serbian version of the Brief Illness Perception Questionnaire (B-IPQ). Psychological status was assessed using the Depression, Anxiety and Stress Scale – 21 items (DASS-21), and quality of life with the WHOQOL-BREF. Sociodemographic and pregnancy-related variables (age, marital status, education, employment, gestational age, parity, history of miscarriages or abortions, previous Cesarean section, comorbidities) were self-reported and verified through medical records. COVID-19–related perceptions were measured through self-report questions regarding fear and adequacy of information. The same instruments and procedures were applied to all participants. |
| Bias | 9 | Describe any efforts to address potential sources of bias  ✓ All patients hospitalized during the study period were invited to participate, with no age-related exclusion criteria, which reduced the risk of selection bias. Participation was voluntary, and informed oral information was provided to ensure that patients could make an autonomous decision, helping to limit coercion-related bias. Moreover, to reduce reporting and social desirability bias, the questionnaire was anonymous, allowing participants to respond more openly about sensitive issues such as mental health, vaccination attitudes, and illness perceptions. |
| Study size | 10 | Explain how the study size was arrived at  ✓The sample size estimation was based on the assumption needed to be fulfilled for the application of factor analysis, set by Tabachnick and Fidell (26), where the minimum number of respondents must be 150, with at least 5 respondents for each item. |
| Quantitative variables | 11 | Explain how quantitative variables were handled in the analyses. If applicable, describe which groupings were chosen and why  ✓Quantitative variables, such as age, body weight, body height, gestational age, and scale scores (IPQ-B, WHOQOL-BREF, DASS-21), were analyzed using descriptive statistics, including means and confidence intervals, to summarize central tendency and variability. Furthermore, for psychometric evaluation, quantitative scale scores were used in confirmatory factor analysis (CFA) and Item Response Theory (IRT) modeling to assess factorial structure, item parameters, and reliability. In path analysis, continuous variables were standardized to allow direct comparison of regression coefficients across different measures, ensuring that effects were expressed on a common scale from −1 to 1. No subgroup or interaction analyses were performed. |
| Statistical methods | 12 | (*a*) Describe all statistical methods, including those used to control for confounding  ✓Descriptive statistics were used to characterize the study sample: means and confidence intervals for numerical variables, and frequencies and percentages for categorical variables. Reliability was evaluated using Cronbach’s α and McDonald’s ω. Confirmatory factor analysis (CFA) was performed to confirm the original two-dimensional structure of the IP. Item Response Theory (IRT) was applied using the graded response model (mirt package in R), with evaluation of item parameters, threshold ordering, S-X² item-fit statistics, and test information/reliability functions. Path analysis was applied to assess direct and indirect effects of predictors by simultaneously modeling interrelated regression relationships. |
|  |  | (*b*) Describe any methods used to examine subgroups and interactions  ✓No subgroup or interaction analyses were performed. |
|  |  | (*c*) Explain how missing data were addressed  ✓Incomplete questionnaires were excluded from the analyses (listwise deletion). |
|  |  | (*d*) If applicable, describe analytical methods taking account of sampling strategy  ✓The entire convenience sample of hospitalized pregnant women was used; no weighting or complex sampling adjustments were required. |
|  |  | (*e*) Describe any sensitivity analyses  ✓No sensitivity analyses were conducted. |
| Results | | |
| Participants | 13* | (a) Report numbers of individuals at each stage of study—eg numbers potentially eligible, examined for eligibility, confirmed eligible, included in the study, completing follow-up, and analysed  ✓All hospitalized pregnant women admitted to the high-risk pregnancy unit during the study period were invited to participate. A total of 290 women completed the questionnaire and were included in the final analysis. |
|  |  | (b) Give reasons for non-participation at each stage  ✓Women who did not provide informed consent or who returned incomplete questionnaires were excluded. |
|  |  | (c) Consider use of a flow diagram  ✓A flow diagram was not used, as all participants who provided complete data were included in the analysis. |
| Descriptive data | 14* | (a) Give characteristics of study participants (eg demographic, clinical, social) and information on exposures and potential confounders.  ✓A total of 290 women with high-risk pregnancies completed the Serbian version of the IPQ. The mean age of study participants was 31.4, with a range of 16–49, while the mean gestational age was 30.0 weeks with a range of 8–41. Most participants (89.3%) had a partner. The majority of the participants had secondary education or below (53.8%). More than half of participants (72.8%) were employed. See Table 1. |
|  |  | (b) Indicate number of participants with missing data for each variable of interest  ✓Only completed questionnaires were included in the analysis; therefore, no missing data were present for the variables reported. |
| Outcome data | 15* | Report numbers of outcome events or summary measures  ✓Mean DASS-21 scores were 3.55 (95% CI 3.10–4.00) for stress, 4.39 (95% CI 3.91–4.86) for anxiety, and 3.99 (95% CI 3.56–4.42) for depression. Mean WHOQOL-BREF subscale scores were 15.92 (95% CI 15.59–16.24) for physical health, 16.13 (95% CI 15.83–16.42) for mental health, 16.86 (95% CI 16.50–17.22) for social relationships, and 15.74 (95% CI 15.43–16.06) for environmental health. For illness perception (IPQ-B), illness concern had the highest mean score (3.83; 95% CI 3.43–4.24), while personal control had the lowest (2.44; 95% CI 2.10–2.78). The reliability of the Serbian IPQ-B was supported with Cronbach’s alpha of 0.70 and McDonald’s omega of 0.85. Path analysis showed that illness perception, depression, anxiety, and stress were the main direct predictors of quality of life |
| Main results | 16 | (*a*) Give unadjusted estimates and, if applicable, confounder-adjusted estimates and their precision (eg, 95% confidence interval). Make clear which confounders were adjusted for and why they were included  ✓Unadjusted estimates (means and 95% CIs) were reported for DASS-21 subscales, WHOQOL-BREF domains, and IPQ-B items. See Table 1 and Table 4. Correlations (Pearson’s r) between illness perception and quality of life domains, as well as between illness perception and DASS-21 scores, were presented with p-values. See Table 2 and 3. Confounder-adjusted estimates were obtained through path analysis, where variables such as marital status, hypertension in pregnancy, depressive and anxiety symptoms, fear for health during COVID-19, and adequacy of information during COVID-19 were included as predictors based on clinical and theoretical relevance. |
|  |  | (*b*) Report category boundaries when continuous variables were categorized  ✓ Continuous variables were analyzed in their original metric form to preserve statistical power so no arbitrary category boundaries were applied. |
|  |  | (*c*) If relevant, consider translating estimates of relative risk into absolute risk for a meaningful time period  ✓ No relative risk of clinical outcomes were calculated so translation into absolute risks was not applicable. |
| Other analyses | 17 | Report other analyses done—eg analyses of subgroups and interactions, and sensitivity analyses  ✓ Subgroup or interaction analyses were not performed, but indirect effects were assessed through path analysis. See Figure 1 and 2. |
| Discussion | | |
| Key results | 18 | Summarise key results with reference to study objectives  ✓ This study examined the validity and reliability of the Serbian version of B-IPQ in the population of women with high-risk pregnancies. The study showed that the Serbian version of this questionnaire had good reliability and validity, and could be used for the illness perception in high-risk pregnancy. Our model demonstrated that illness perception was one of the main direct predictors of quality of life, as well as an indirect predictor through depression, anxiety and stress levels. Marital status, hypertension in pregnancy, fear for health during covid pandemic, and being informed during covid pandemic had direct negative effects on illness perceptions, and therefore on QoL indirectly. |
| Limitations | 19 | Discuss limitations of the study, taking into account sources of potential bias or imprecision. Discuss both direction and magnitude of any potential bias  ✓The primary limitation of this study was that the sample included only hospitalized patients. We limited our sample to hospitalized patients to ensure a controlled environment for data collection and to focus on cases where the conditions under investigation were closely monitored. This approach was chosen to minimize variability in patient management and ensure the reliability of our findings in this initial phase of research. As this represents preliminary research on the topic, future studies should be prospective and aim to include high-risk pregnancies managed in outpatient settings to provide a more comprehensive understanding. Moreover, another limitation of our study was a cross-sectional design that was necessary for questionnaire validation. |
| Interpretation | 20 | Give a cautious overall interpretation of results considering objectives, limitations, multiplicity of analyses, results from similar studies, and other relevant evidence  ✓ The study showed that the Serbian version of this questionnaire had good reliability and validity, and could be used for the illness perception in high-risk pregnancy. The primary limitation of this study was that the sample included only hospitalized patients which was done to ensure a controlled environment for data collection and bias reduction. Our results suggest that illness perception correlated significantly with quality of life related to mental health (except perception of illness duration) as well as stress, anxiety, and depression levels. The consequence domain of illness perception affected mental health mostly similarly to an Australian study (see reference 32), This can probably be explained by the future mother’s worry for the baby. Relevant to this, Scime et al.(38) showed that worse illness perception in pregnant women with chronic disease decreased their motivation for breastfeeding, a great example of how illness perception can have more profound personal and public health consequences. |
| Generalisability | 21 | Discuss the generalisability (external validity) of the study results  ✓ The study showed that the Serbian version of this questionnaire had good reliability and validity, and could be used for the illness perception in high-risk pregnancy. Implementing this brief and time-efficient questionnaire in patients with high-risk pregnancies could help identify those with maladaptive illness perceptions, prompting clinicians to provide appropriate reassurance and interventions to improve their quality of life. Additionally, it may detect patients who are reluctant to discuss depression or anxiety due to stigma but are more comfortable addressing pregnancy-related concerns, enabling obstetricians to refer them to mental health professionals when necessary |
| Other information | | |
| Funding | 22 | Give the source of funding and the role of the funders for the present study and, if applicable, for the original study on which the present article is based.  ✓ No funding to disclose. |

*Give information separately for exposed and unexposed groups.

**Note:** An Explanation and Elaboration article discusses each checklist item and gives methodological background and published examples of transparent reporting. The STROBE checklist is best used in conjunction with this article (freely available on the Web sites of PLoS Medicine at http://www.plosmedicine.org/, Annals of Internal Medicine at http://www.annals.org/, and Epidemiology at http://www.epidem.com/). Information on the STROBE Initiative is available at www.strobe-statement.org.
