# Supplementary material for: First Balkan Brief Illness Perception Questionnaire (IPQ-B) among high-risk pregnancies
Source: PLoS One. 2025 Oct 28;20(10):e0334844. doi: 10.1371/journal.pone.0334844 (PMC12561911; doi:10.1371/journal.pone.0334844)
Supplement: S7 File — (AMOSOUTPUT) [file pone.0334844.s007.AmosOutput]

maja konfirmatorna sva pitanja recenzija.amw


#### C:\Users\Admin\Desktop\Maja Macura\maja konfirmatorna sva pitanja recenzija.amw

##### Analysis Summary

##### Date and Time

Date: sreda, 05. mart 2025.

Time: 16:29:07

##### Title

maja konfirmatorna sva pitanja recenzija: sreda, 05. mart 2025. 16:29

##### Groups

##### Group number 1 (Group number 1)

##### Notes for Group (Group number 1)

The model is recursive.

Sample size = 290

##### Variable Summary (Group number 1)

##### Your model contains the following variables (Group number 1)

Observed, endogenous variables

IP1

IP2

IP3

IP4

IP5

IP6

IP7

IP8

Unobserved, exogenous variables

cognitive

e1

e2

e3

e4

e5

emotional

e6

e7

e8

##### Variable counts (Group number 1)

|  |  |
| --- | --- |
| Number of variables in your model: | 18 |
| Number of observed variables: | 8 |
| Number of unobserved variables: | 10 |
| Number of exogenous variables: | 10 |
| Number of endogenous variables: | 8 |

##### Parameter Summary (Group number 1)

|  | Weights | Covariances | Variances | Means | Intercepts | Total |
| --- | --- | --- | --- | --- | --- | --- |
| Fixed | 10 | 0 | 0 | 0 | 0 | 10 |
| Labeled | 0 | 0 | 0 | 0 | 0 | 0 |
| Unlabeled | 6 | 1 | 10 | 0 | 8 | 25 |
| Total | 16 | 1 | 10 | 0 | 8 | 35 |

##### Models

##### Default model (Default model)

##### Notes for Model (Default model)

##### Computation of degrees of freedom (Default model)

|  |  |
| --- | --- |
| Number of distinct sample moments: | 44 |
| Number of distinct parameters to be estimated: | 25 |
| Degrees of freedom (44 - 25): | 19 |

##### Result (Default model)

Minimum was achieved

Chi-square = 203,156

Degrees of freedom = 19

Probability level = ,000

##### Group number 1 (Group number 1 - Default model)

##### Estimates (Group number 1 - Default model)

##### Scalar Estimates (Group number 1 - Default model)

##### Maximum Likelihood Estimates

##### Regression Weights: (Group number 1 - Default model)

|  |  |  | Estimate | S.E. | C.R. | P | Label |
| --- | --- | --- | --- | --- | --- | --- | --- |
| IP1 | <--- | cognitive | 1,000 |  |
| IP2 | <--- | cognitive | ,814 | ,084 | 9,672 | \*\*\* |  |
| IP3 | <--- | cognitive | ,756 | ,059 | 12,741 | \*\*\* |  |
| IP4 | <--- | cognitive | -,276 | ,083 | -3,316 | \*\*\* |  |
| IP5 | <--- | cognitive | ,877 | ,059 | 14,834 | \*\*\* |  |
| IP6 | <--- | emotional | 1,000 |  |
| IP7 | <--- | emotional | -,293 | ,090 | -3,261 | ,001 |  |
| IP8 | <--- | emotional | ,979 | ,078 | 12,605 | \*\*\* |  |

##### Standardized Regression Weights: (Group number 1 - Default model)

|  |  |  | Estimate |
| --- | --- | --- | --- |
| IP1 | <--- | cognitive | ,866 |
| IP2 | <--- | cognitive | ,552 |
| IP3 | <--- | cognitive | ,688 |
| IP4 | <--- | cognitive | -,205 |
| IP5 | <--- | cognitive | ,771 |
| IP6 | <--- | emotional | ,747 |
| IP7 | <--- | emotional | -,207 |
| IP8 | <--- | emotional | ,797 |

##### Intercepts: (Group number 1 - Default model)

|  |  |  | Estimate | S.E. | C.R. | P | Label |
| --- | --- | --- | --- | --- | --- | --- | --- |
| IP1 |  |  | 2,648 | ,182 | 14,567 | \*\*\* |  |
| IP2 |  |  | 3,355 | ,232 | 14,460 | \*\*\* |  |
| IP3 |  |  | 2,438 | ,173 | 14,100 | \*\*\* |  |
| IP4 |  |  | 2,962 | ,212 | 13,958 | \*\*\* |  |
| IP5 |  |  | 2,559 | ,179 | 14,278 | \*\*\* |  |
| IP6 |  |  | 3,834 | ,204 | 18,828 | \*\*\* |  |
| IP7 |  |  | 3,228 | ,215 | 14,995 | \*\*\* |  |
| IP8 |  |  | 2,790 | ,187 | 14,919 | \*\*\* |  |

##### Covariances: (Group number 1 - Default model)

|  |  |  | Estimate | S.E. | C.R. | P | Label |
| --- | --- | --- | --- | --- | --- | --- | --- |
| emotional | <--> | cognitive | 6,478 | ,724 | 8,954 | \*\*\* |  |

##### Correlations: (Group number 1 - Default model)

|  |  |  | Estimate |
| --- | --- | --- | --- |
| emotional | <--> | cognitive | ,936 |

##### Variances: (Group number 1 - Default model)

|  |  |  | Estimate | S.E. | C.R. | P | Label |
| --- | --- | --- | --- | --- | --- | --- | --- |
| cognitive |  |  | 7,157 | ,815 | 8,782 | \*\*\* |  |
| emotional |  |  | 6,691 | ,969 | 6,903 | \*\*\* |  |
| e1 |  |  | 2,395 | ,335 | 7,149 | \*\*\* |  |
| e2 |  |  | 10,820 | ,955 | 11,334 | \*\*\* |  |
| e3 |  |  | 4,552 | ,430 | 10,591 | \*\*\* |  |
| e4 |  |  | 12,470 | 1,043 | 11,953 | \*\*\* |  |
| e5 |  |  | 3,771 | ,391 | 9,644 | \*\*\* |  |
| e6 |  |  | 5,295 | ,577 | 9,173 | \*\*\* |  |
| e7 |  |  | 12,817 | 1,074 | 11,931 | \*\*\* |  |
| e8 |  |  | 3,686 | ,470 | 7,843 | \*\*\* |  |

##### Matrices (Group number 1 - Default model)

##### Residual Covariances (Group number 1 - Default model)

|  | IP8 | IP7 | IP6 | IP5 | IP4 | IP3 | IP2 | IP1 |
| --- | --- | --- | --- | --- | --- | --- | --- | --- |
| IP8 | ,000 |
| IP7 | ,944 | ,000 |
| IP6 | ,033 | -,946 | ,000 |
| IP5 | -,108 | -,040 | -,047 | ,000 |
| IP4 | ,372 | 8,020 | -1,520 | ,704 | ,000 |
| IP3 | ,450 | ,536 | ,133 | -,069 | ,745 | ,000 |
| IP2 | -1,119 | -1,914 | -,385 | -,140 | -1,985 | ,805 | ,000 |
| IP1 | ,168 | ,272 | -,167 | ,148 | ,253 | -,275 | ,166 | ,000 |

##### Residual Means (Group number 1 - Default model)

|  | IP8 | IP7 | IP6 | IP5 | IP4 | IP3 | IP2 | IP1 |
| --- | --- | --- | --- | --- | --- | --- | --- | --- |
|  | ,000 | ,000 | ,000 | ,000 | ,000 | ,000 | ,000 | ,000 |

##### Standardized Residual Covariances (Group number 1 - Default model)

|  | IP8 | IP7 | IP6 | IP5 | IP4 | IP3 | IP2 | IP1 |
| --- | --- | --- | --- | --- | --- | --- | --- | --- |
| IP8 | ,000 |
| IP7 | 1,362 | ,000 |
| IP6 | ,044 | -1,255 | ,000 |
| IP5 | -,164 | -,060 | -,066 | ,000 |
| IP4 | ,545 | 10,319 | -2,048 | 1,076 | ,000 |
| IP3 | ,728 | ,840 | ,200 | -,116 | 1,183 | ,000 |
| IP2 | -1,403 | -2,242 | -,447 | -,182 | -2,356 | 1,104 | ,000 |
| IP1 | ,244 | ,403 | -,227 | ,222 | ,380 | -,442 | ,209 | ,000 |

##### Standardized Residual Means (Group number 1 - Default model)

|  | IP8 | IP7 | IP6 | IP5 | IP4 | IP3 | IP2 | IP1 |
| --- | --- | --- | --- | --- | --- | --- | --- | --- |
|  | ,000 | ,000 | ,000 | ,000 | ,000 | ,000 | ,000 | ,000 |

##### Modification Indices (Group number 1 - Default model)

##### Covariances: (Group number 1 - Default model)

|  |  |  | M.I. | Par Change |
| --- | --- | --- | --- | --- |
| e7 | <--> | e8 | 7,287 | 1,261 |
| e6 | <--> | e7 | 4,672 | -1,160 |
| e4 | <--> | cognitive | 5,066 | ,896 |
| e4 | <--> | emotional | 6,561 | -1,050 |
| e4 | <--> | e7 | 115,189 | 8,027 |
| e4 | <--> | e6 | 9,130 | -1,599 |
| e2 | <--> | cognitive | 4,612 | ,805 |
| e2 | <--> | emotional | 6,035 | -,954 |
| e2 | <--> | e8 | 8,875 | -1,305 |
| e2 | <--> | e7 | 8,120 | -2,032 |
| e2 | <--> | e4 | 8,922 | -2,099 |
| e2 | <--> | e3 | 4,436 | ,939 |

##### Variances: (Group number 1 - Default model)

|  |  |  | M.I. | Par Change |
| --- | --- | --- | --- | --- |

##### Regression Weights: (Group number 1 - Default model)

|  |  |  | M.I. | Par Change |
| --- | --- | --- | --- | --- |
| IP7 | <--- | IP4 | 65,894 | ,367 |
| IP6 | <--- | IP4 | 5,140 | -,073 |
| IP4 | <--- | IP7 | 62,638 | ,338 |
| IP2 | <--- | IP7 | 4,088 | -,082 |
| IP2 | <--- | IP4 | 5,082 | -,096 |

##### Means: (Group number 1 - Default model)

|  |  |  | M.I. | Par Change |
| --- | --- | --- | --- | --- |

##### Intercepts: (Group number 1 - Default model)

|  |  |  | M.I. | Par Change |
| --- | --- | --- | --- | --- |

##### Minimization History (Default model)

| Iteration |  | Negative eigenvalues | Condition # | Smallest eigenvalue | Diameter | F | NTries | Ratio |
| --- | --- | --- | --- | --- | --- | --- | --- | --- |
| 0 | e | 4 |  | -,451 | 9999,000 | 1043,924 | 0 | 9999,000 |
| 1 | e\* | 2 |  | -,262 | 2,023 | 406,777 | 20 | ,518 |
| 2 | e | 1 |  | -,002 | ,222 | 317,715 | 7 | ,914 |
| 3 | e | 0 | 416,737 |  | ,585 | 223,117 | 7 | ,748 |
| 4 | e | 0 | 124,322 |  | ,439 | 205,988 | 2 | ,000 |
| 5 | e | 0 | 98,298 |  | ,077 | 203,225 | 1 | 1,069 |
| 6 | e | 0 | 94,798 |  | ,016 | 203,156 | 1 | 1,028 |
| 7 | e | 0 | 94,484 |  | ,001 | 203,156 | 1 | 1,002 |

##### Model Fit Summary

##### CMIN

| Model | NPAR | CMIN | DF | P | CMIN/DF |
| --- | --- | --- | --- | --- | --- |
| Default model | 25 | 203,156 | 19 | ,000 | 10,692 |
| Saturated model | 44 | ,000 | 0 |
| Independence model | 16 | 985,913 | 28 | ,000 | 35,211 |

##### Baseline Comparisons

| Model | NFI Delta1 | RFI rho1 | IFI Delta2 | TLI rho2 | CFI |
| --- | --- | --- | --- | --- | --- |
| Default model | ,794 | ,696 | ,810 | ,717 | ,808 |
| Saturated model | 1,000 |  | 1,000 |  | 1,000 |
| Independence model | ,000 | ,000 | ,000 | ,000 | ,000 |

##### Parsimony-Adjusted Measures

| Model | PRATIO | PNFI | PCFI |
| --- | --- | --- | --- |
| Default model | ,679 | ,539 | ,548 |
| Saturated model | ,000 | ,000 | ,000 |
| Independence model | 1,000 | ,000 | ,000 |

##### NCP

| Model | NCP | LO 90 | HI 90 |
| --- | --- | --- | --- |
| Default model | 184,156 | 142,048 | 233,724 |
| Saturated model | ,000 | ,000 | ,000 |
| Independence model | 957,913 | 859,014 | 1064,209 |

##### FMIN

| Model | FMIN | F0 | LO 90 | HI 90 |
| --- | --- | --- | --- | --- |
| Default model | ,703 | ,637 | ,492 | ,809 |
| Saturated model | ,000 | ,000 | ,000 | ,000 |
| Independence model | 3,411 | 3,315 | 2,972 | 3,682 |

##### RMSEA

| Model | RMSEA | LO 90 | HI 90 | PCLOSE |
| --- | --- | --- | --- | --- |
| Default model | ,183 | ,161 | ,206 | ,000 |
| Independence model | ,344 | ,326 | ,363 | ,000 |

##### AIC

| Model | AIC | BCC | BIC | CAIC |
| --- | --- | --- | --- | --- |
| Default model | 253,156 | 254,763 |
| Saturated model | 88,000 | 90,829 |
| Independence model | 1017,913 | 1018,942 |

##### ECVI

| Model | ECVI | LO 90 | HI 90 | MECVI |
| --- | --- | --- | --- | --- |
| Default model | ,876 | ,730 | 1,047 | ,882 |
| Saturated model | ,304 | ,304 | ,304 | ,314 |
| Independence model | 3,522 | 3,180 | 3,890 | 3,526 |

##### HOELTER

| Model | HOELTER .05 | HOELTER .01 |
| --- | --- | --- |
| Default model | 43 | 52 |
| Independence model | 13 | 15 |

##### Execution time summary

|  |  |
| --- | --- |
| Minimization: | ,023 |
| Miscellaneous: | ,224 |
| Bootstrap: | ,000 |
| Total: | ,247 |
