# Supplementary material for: First Balkan Brief Illness Perception Questionnaire (IPQ-B) among high-risk pregnancies
Source: PLoS One. 2025 Oct 28;20(10):e0334844. doi: 10.1371/journal.pone.0334844 (PMC12561911; doi:10.1371/journal.pone.0334844)
Supplement: S11 File — (AMOSOUTPUT) [file pone.0334844.s011.AmosOutput]

Maja path novo.amw


#### C:\Users\Admin\Desktop\Maja Macura\Path\Maja path novo.amw

##### Analysis Summary

##### Date and Time

Date: utorak, 27. jun 2023.

Time: 17:36:11

##### Title

Maja path novo: utorak, 27. jun 2023. 17:36

##### Groups

##### Group number 1 (Group number 1)

##### Notes for Group (Group number 1)

The model is recursive.

Sample size = 290

##### Variable Summary (Group number 1)

##### Your model contains the following variables (Group number 1)

Observed, endogenous variables

TOTALIP

WHOQOL\_DOM2\_PSYCHOLOGICAL

Observed, exogenous variables

T\_bracni\_status\_1

HTA

COV2\_1

COV3\_1

ttDASS21DEPRESIJA

ttDASS21ANKSIOZNOST

ttDASS21STRES

COV4\_1

T\_zaposlena\_1

Unobserved, exogenous variables

e1

e2

##### Variable counts (Group number 1)

|  |  |
| --- | --- |
| Number of variables in your model: | 13 |
| Number of observed variables: | 11 |
| Number of unobserved variables: | 2 |
| Number of exogenous variables: | 11 |
| Number of endogenous variables: | 2 |

##### Parameter Summary (Group number 1)

|  | Weights | Covariances | Variances | Means | Intercepts | Total |
| --- | --- | --- | --- | --- | --- | --- |
| Fixed | 2 | 0 | 0 | 0 | 0 | 2 |
| Labeled | 0 | 0 | 0 | 0 | 0 | 0 |
| Unlabeled | 10 | 19 | 11 | 0 | 0 | 40 |
| Total | 12 | 19 | 11 | 0 | 0 | 42 |

##### Models

##### Default model (Default model)

##### Notes for Model (Default model)

##### Computation of degrees of freedom (Default model)

|  |  |
| --- | --- |
| Number of distinct sample moments: | 66 |
| Number of distinct parameters to be estimated: | 40 |
| Degrees of freedom (66 - 40): | 26 |

##### Result (Default model)

Minimum was achieved

Chi-square = 36,105

Degrees of freedom = 26

Probability level = ,090

##### Group number 1 (Group number 1 - Default model)

##### Estimates (Group number 1 - Default model)

##### Scalar Estimates (Group number 1 - Default model)

##### Maximum Likelihood Estimates

##### Regression Weights: (Group number 1 - Default model)

|  |  |  | Estimate | S.E. | C.R. | P | Label |
| --- | --- | --- | --- | --- | --- | --- | --- |
| TOTALIP | <--- | T\_bracni\_status\_1 | -7,599 | 2,508 | -3,030 | ,002 | par\_1 |
| TOTALIP | <--- | HTA | 5,320 | 2,103 | 2,529 | ,011 | par\_2 |
| TOTALIP | <--- | COV2\_1 | 6,453 | 1,674 | 3,855 | \*\*\* | par\_3 |
| TOTALIP | <--- | COV3\_1 | -5,856 | 1,642 | -3,566 | \*\*\* | par\_4 |
| TOTALIP | <--- | ttDASS21DEPRESIJA | 5,837 | 1,888 | 3,092 | ,002 | par\_5 |
| TOTALIP | <--- | ttDASS21ANKSIOZNOST | 5,172 | 1,747 | 2,962 | ,003 | par\_6 |
| WHOQOL\_DOM2\_PSYCHOLOGICAL | <--- | ttDASS21DEPRESIJA | -,931 | ,322 | -2,892 | ,004 | par\_7 |
| WHOQOL\_DOM2\_PSYCHOLOGICAL | <--- | ttDASS21ANKSIOZNOST | -,993 | ,287 | -3,459 | \*\*\* | par\_8 |
| WHOQOL\_DOM2\_PSYCHOLOGICAL | <--- | ttDASS21STRES | -1,986 | ,389 | -5,101 | \*\*\* | par\_9 |
| WHOQOL\_DOM2\_PSYCHOLOGICAL | <--- | TOTALIP | -,030 | ,009 | -3,491 | \*\*\* | par\_10 |

##### Standardized Regression Weights: (Group number 1 - Default model)

|  |  |  | Estimate |
| --- | --- | --- | --- |
| TOTALIP | <--- | T\_bracni\_status\_1 | -,157 |
| TOTALIP | <--- | HTA | ,128 |
| TOTALIP | <--- | COV2\_1 | ,200 |
| TOTALIP | <--- | COV3\_1 | -,181 |
| TOTALIP | <--- | ttDASS21DEPRESIJA | ,184 |
| TOTALIP | <--- | ttDASS21ANKSIOZNOST | ,175 |
| WHOQOL\_DOM2\_PSYCHOLOGICAL | <--- | ttDASS21DEPRESIJA | -,170 |
| WHOQOL\_DOM2\_PSYCHOLOGICAL | <--- | ttDASS21ANKSIOZNOST | -,194 |
| WHOQOL\_DOM2\_PSYCHOLOGICAL | <--- | ttDASS21STRES | -,292 |
| WHOQOL\_DOM2\_PSYCHOLOGICAL | <--- | TOTALIP | -,173 |

##### Covariances: (Group number 1 - Default model)

|  |  |  | Estimate | S.E. | C.R. | P | Label |
| --- | --- | --- | --- | --- | --- | --- | --- |
| ttDASS21ANKSIOZNOST | <--> | ttDASS21STRES | ,089 | ,012 | 7,520 | \*\*\* | par\_11 |
| ttDASS21DEPRESIJA | <--> | ttDASS21ANKSIOZNOST | ,115 | ,015 | 7,684 | \*\*\* | par\_12 |
| ttDASS21DEPRESIJA | <--> | ttDASS21STRES | ,095 | ,011 | 8,265 | \*\*\* | par\_13 |
| ttDASS21STRES | <--> | COV4\_1 | ,030 | ,008 | 3,891 | \*\*\* | par\_14 |
| T\_bracni\_status\_1 | <--> | T\_zaposlena\_1 | ,025 | ,008 | 3,113 | ,002 | par\_15 |
| COV2\_1 | <--> | ttDASS21STRES | ,019 | ,009 | 2,057 | ,040 | par\_16 |
| ttDASS21STRES | <--> | T\_zaposlena\_1 | -,034 | ,009 | -3,939 | \*\*\* | par\_17 |
| T\_bracni\_status\_1 | <--> | ttDASS21STRES | -,032 | ,007 | -4,678 | \*\*\* | par\_18 |
| COV3\_1 | <--> | COV4\_1 | -,014 | ,009 | -1,524 | ,128 | par\_19 |
| T\_bracni\_status\_1 | <--> | ttDASS21DEPRESIJA | -,025 | ,008 | -3,080 | ,002 | par\_20 |
| ttDASS21DEPRESIJA | <--> | T\_zaposlena\_1 | -,031 | ,010 | -2,981 | ,003 | par\_21 |
| COV2\_1 | <--> | ttDASS21DEPRESIJA | ,040 | ,012 | 3,243 | ,001 | par\_22 |
| ttDASS21ANKSIOZNOST | <--> | COV4\_1 | ,021 | ,010 | 2,123 | ,034 | par\_23 |
| COV2\_1 | <--> | ttDASS21ANKSIOZNOST | ,038 | ,013 | 2,856 | ,004 | par\_24 |
| T\_bracni\_status\_1 | <--> | ttDASS21ANKSIOZNOST | -,021 | ,009 | -2,471 | ,013 | par\_25 |
| ttDASS21DEPRESIJA | <--> | COV4\_1 | ,020 | ,009 | 2,136 | ,033 | par\_26 |
| COV3\_1 | <--> | T\_zaposlena\_1 | ,020 | ,011 | 1,778 | ,075 | par\_27 |
| T\_bracni\_status\_1 | <--> | COV4\_1 | -,022 | ,006 | -3,393 | \*\*\* | par\_28 |
| COV4\_1 | <--> | T\_zaposlena\_1 | -,021 | ,009 | -2,307 | ,021 | par\_29 |

##### Correlations: (Group number 1 - Default model)

|  |  |  | Estimate |
| --- | --- | --- | --- |
| ttDASS21ANKSIOZNOST | <--> | ttDASS21STRES | ,481 |
| ttDASS21DEPRESIJA | <--> | ttDASS21ANKSIOZNOST | ,500 |
| ttDASS21DEPRESIJA | <--> | ttDASS21STRES | ,546 |
| ttDASS21STRES | <--> | COV4\_1 | ,232 |
| T\_bracni\_status\_1 | <--> | T\_zaposlena\_1 | ,183 |
| COV2\_1 | <--> | ttDASS21STRES | ,114 |
| ttDASS21STRES | <--> | T\_zaposlena\_1 | -,208 |
| T\_bracni\_status\_1 | <--> | ttDASS21STRES | -,282 |
| COV3\_1 | <--> | COV4\_1 | -,087 |
| T\_bracni\_status\_1 | <--> | ttDASS21DEPRESIJA | -,180 |
| ttDASS21DEPRESIJA | <--> | T\_zaposlena\_1 | -,152 |
| COV2\_1 | <--> | ttDASS21DEPRESIJA | ,189 |
| ttDASS21ANKSIOZNOST | <--> | COV4\_1 | ,123 |
| COV2\_1 | <--> | ttDASS21ANKSIOZNOST | ,168 |
| T\_bracni\_status\_1 | <--> | ttDASS21ANKSIOZNOST | -,142 |
| ttDASS21DEPRESIJA | <--> | COV4\_1 | ,124 |
| COV3\_1 | <--> | T\_zaposlena\_1 | ,101 |
| T\_bracni\_status\_1 | <--> | COV4\_1 | -,203 |
| COV4\_1 | <--> | T\_zaposlena\_1 | -,136 |

##### Variances: (Group number 1 - Default model)

|  |  |  | Estimate | S.E. | C.R. | P | Label |
| --- | --- | --- | --- | --- | --- | --- | --- |
| T\_bracni\_status\_1 |  |  | ,092 | ,008 | 12,029 | \*\*\* | par\_30 |
| HTA |  |  | ,126 | ,011 | 12,021 | \*\*\* | par\_31 |
| COV2\_1 |  |  | ,209 | ,017 | 12,021 | \*\*\* | par\_32 |
| COV3\_1 |  |  | ,207 | ,017 | 12,021 | \*\*\* | par\_33 |
| ttDASS21DEPRESIJA |  |  | ,216 | ,018 | 12,108 | \*\*\* | par\_34 |
| ttDASS21ANKSIOZNOST |  |  | ,247 | ,021 | 12,031 | \*\*\* | par\_35 |
| ttDASS21STRES |  |  | ,139 | ,011 | 12,159 | \*\*\* | par\_36 |
| COV4\_1 |  |  | ,123 | ,010 | 12,031 | \*\*\* | par\_37 |
| T\_zaposlena\_1 |  |  | ,194 | ,016 | 12,031 | \*\*\* | par\_38 |
| e1 |  |  | 161,445 | 13,430 | 12,021 | \*\*\* | par\_39 |
| e2 |  |  | 3,926 | ,327 | 12,021 | \*\*\* | par\_40 |

##### Squared Multiple Correlations: (Group number 1 - Default model)

|  |  |  | Estimate |
| --- | --- | --- | --- |
| TOTALIP |  |  | ,255 |
| WHOQOL\_DOM2\_PSYCHOLOGICAL |  |  | ,391 |

##### Matrices (Group number 1 - Default model)

##### Total Effects (Group number 1 - Default model)

|  | ttDASS21STRES | ttDASS21ANKSIOZNOST | ttDASS21DEPRESIJA | COV3\_1 | COV2\_1 | HTA | T\_bracni\_status\_1 | TOTALIP |
| --- | --- | --- | --- | --- | --- | --- | --- | --- |
| TOTALIP | ,000 | 5,172 | 5,837 | -5,856 | 6,453 | 5,320 | -7,599 | ,000 |
| WHOQOL\_DOM2\_PSYCHOLOGICAL | -1,986 | -1,147 | -1,106 | ,175 | -,193 | -,159 | ,227 | -,030 |

##### Standardized Total Effects (Group number 1 - Default model)

|  | ttDASS21STRES | ttDASS21ANKSIOZNOST | ttDASS21DEPRESIJA | COV3\_1 | COV2\_1 | HTA | T\_bracni\_status\_1 | TOTALIP |
| --- | --- | --- | --- | --- | --- | --- | --- | --- |
| TOTALIP | ,000 | ,175 | ,184 | -,181 | ,200 | ,128 | -,157 | ,000 |
| WHOQOL\_DOM2\_PSYCHOLOGICAL | -,292 | -,225 | -,202 | ,031 | -,035 | -,022 | ,027 | -,173 |

##### Direct Effects (Group number 1 - Default model)

|  | ttDASS21STRES | ttDASS21ANKSIOZNOST | ttDASS21DEPRESIJA | COV3\_1 | COV2\_1 | HTA | T\_bracni\_status\_1 | TOTALIP |
| --- | --- | --- | --- | --- | --- | --- | --- | --- |
| TOTALIP | ,000 | 5,172 | 5,837 | -5,856 | 6,453 | 5,320 | -7,599 | ,000 |
| WHOQOL\_DOM2\_PSYCHOLOGICAL | -1,986 | -,993 | -,931 | ,000 | ,000 | ,000 | ,000 | -,030 |

##### Standardized Direct Effects (Group number 1 - Default model)

|  | ttDASS21STRES | ttDASS21ANKSIOZNOST | ttDASS21DEPRESIJA | COV3\_1 | COV2\_1 | HTA | T\_bracni\_status\_1 | TOTALIP |
| --- | --- | --- | --- | --- | --- | --- | --- | --- |
| TOTALIP | ,000 | ,175 | ,184 | -,181 | ,200 | ,128 | -,157 | ,000 |
| WHOQOL\_DOM2\_PSYCHOLOGICAL | -,292 | -,194 | -,170 | ,000 | ,000 | ,000 | ,000 | -,173 |

##### Indirect Effects (Group number 1 - Default model)

|  | ttDASS21STRES | ttDASS21ANKSIOZNOST | ttDASS21DEPRESIJA | COV3\_1 | COV2\_1 | HTA | T\_bracni\_status\_1 | TOTALIP |
| --- | --- | --- | --- | --- | --- | --- | --- | --- |
| TOTALIP | ,000 | ,000 | ,000 | ,000 | ,000 | ,000 | ,000 | ,000 |
| WHOQOL\_DOM2\_PSYCHOLOGICAL | ,000 | -,155 | -,175 | ,175 | -,193 | -,159 | ,227 | ,000 |

##### Standardized Indirect Effects (Group number 1 - Default model)

|  | ttDASS21STRES | ttDASS21ANKSIOZNOST | ttDASS21DEPRESIJA | COV3\_1 | COV2\_1 | HTA | T\_bracni\_status\_1 | TOTALIP |
| --- | --- | --- | --- | --- | --- | --- | --- | --- |
| TOTALIP | ,000 | ,000 | ,000 | ,000 | ,000 | ,000 | ,000 | ,000 |
| WHOQOL\_DOM2\_PSYCHOLOGICAL | ,000 | -,030 | -,032 | ,031 | -,035 | -,022 | ,027 | ,000 |

##### Modification Indices (Group number 1 - Default model)

##### Covariances: (Group number 1 - Default model)

|  |  |  | M.I. | Par Change |
| --- | --- | --- | --- | --- |

##### Variances: (Group number 1 - Default model)

|  |  |  | M.I. | Par Change |
| --- | --- | --- | --- | --- |

##### Regression Weights: (Group number 1 - Default model)

|  |  |  | M.I. | Par Change |
| --- | --- | --- | --- | --- |

##### Bootstrap (Group number 1 - Default model)

##### Bootstrap standard errors (Group number 1 - Default model)

##### Scalar Estimates (Group number 1 - Default model)

##### Regression Weights: (Group number 1 - Default model)

| Parameter | | | SE | SE-SE | Mean | Bias | SE-Bias |
| --- | --- | --- | --- | --- | --- | --- | --- |
| TOTALIP | <--- | T\_bracni\_status\_1 | 2,685 | ,134 | -7,770 | -,170 | ,190 |
| TOTALIP | <--- | HTA | 2,510 | ,126 | 5,323 | ,003 | ,177 |
| TOTALIP | <--- | COV2\_1 | 1,859 | ,093 | 6,391 | -,062 | ,131 |
| TOTALIP | <--- | COV3\_1 | 1,780 | ,089 | -5,800 | ,056 | ,126 |
| TOTALIP | <--- | ttDASS21DEPRESIJA | 1,767 | ,088 | 6,040 | ,203 | ,125 |
| TOTALIP | <--- | ttDASS21ANKSIOZNOST | 1,731 | ,087 | 4,969 | -,203 | ,122 |
| WHOQOL\_DOM2\_PSYCHOLOGICAL | <--- | ttDASS21DEPRESIJA | ,386 | ,019 | -,903 | ,028 | ,027 |
| WHOQOL\_DOM2\_PSYCHOLOGICAL | <--- | ttDASS21ANKSIOZNOST | ,277 | ,014 | -1,025 | -,032 | ,020 |
| WHOQOL\_DOM2\_PSYCHOLOGICAL | <--- | ttDASS21STRES | ,451 | ,023 | -1,992 | -,005 | ,032 |
| WHOQOL\_DOM2\_PSYCHOLOGICAL | <--- | TOTALIP | ,010 | ,001 | -,029 | ,001 | ,001 |

##### Standardized Regression Weights: (Group number 1 - Default model)

| Parameter | | | SE | SE-SE | Mean | Bias | SE-Bias |
| --- | --- | --- | --- | --- | --- | --- | --- |
| TOTALIP | <--- | T\_bracni\_status\_1 | ,055 | ,003 | -,160 | -,003 | ,004 |
| TOTALIP | <--- | HTA | ,060 | ,003 | ,128 | -,001 | ,004 |
| TOTALIP | <--- | COV2\_1 | ,057 | ,003 | ,199 | -,001 | ,004 |
| TOTALIP | <--- | COV3\_1 | ,056 | ,003 | -,180 | ,001 | ,004 |
| TOTALIP | <--- | ttDASS21DEPRESIJA | ,054 | ,003 | ,190 | ,006 | ,004 |
| TOTALIP | <--- | ttDASS21ANKSIOZNOST | ,057 | ,003 | ,168 | -,007 | ,004 |
| WHOQOL\_DOM2\_PSYCHOLOGICAL | <--- | ttDASS21DEPRESIJA | ,069 | ,003 | -,165 | ,006 | ,005 |
| WHOQOL\_DOM2\_PSYCHOLOGICAL | <--- | ttDASS21ANKSIOZNOST | ,057 | ,003 | -,202 | -,008 | ,004 |
| WHOQOL\_DOM2\_PSYCHOLOGICAL | <--- | ttDASS21STRES | ,062 | ,003 | -,291 | ,001 | ,004 |
| WHOQOL\_DOM2\_PSYCHOLOGICAL | <--- | TOTALIP | ,059 | ,003 | -,171 | ,003 | ,004 |

##### Covariances: (Group number 1 - Default model)

| Parameter | | | SE | SE-SE | Mean | Bias | SE-Bias |
| --- | --- | --- | --- | --- | --- | --- | --- |
| ttDASS21ANKSIOZNOST | <--> | ttDASS21STRES | ,011 | ,001 | ,088 | -,001 | ,001 |
| ttDASS21DEPRESIJA | <--> | ttDASS21ANKSIOZNOST | ,013 | ,001 | ,115 | -,001 | ,001 |
| ttDASS21DEPRESIJA | <--> | ttDASS21STRES | ,013 | ,001 | ,095 | ,000 | ,001 |
| ttDASS21STRES | <--> | COV4\_1 | ,010 | ,000 | ,029 | -,001 | ,001 |
| T\_bracni\_status\_1 | <--> | T\_zaposlena\_1 | ,009 | ,000 | ,025 | ,000 | ,001 |
| COV2\_1 | <--> | ttDASS21STRES | ,010 | ,000 | ,018 | -,001 | ,001 |
| ttDASS21STRES | <--> | T\_zaposlena\_1 | ,010 | ,000 | -,034 | ,000 | ,001 |
| T\_bracni\_status\_1 | <--> | ttDASS21STRES | ,010 | ,000 | -,031 | ,001 | ,001 |
| COV3\_1 | <--> | COV4\_1 | ,010 | ,000 | -,014 | ,000 | ,001 |
| T\_bracni\_status\_1 | <--> | ttDASS21DEPRESIJA | ,009 | ,000 | -,026 | ,000 | ,001 |
| ttDASS21DEPRESIJA | <--> | T\_zaposlena\_1 | ,010 | ,001 | -,033 | -,002 | ,001 |
| COV2\_1 | <--> | ttDASS21DEPRESIJA | ,013 | ,001 | ,040 | ,000 | ,001 |
| ttDASS21ANKSIOZNOST | <--> | COV4\_1 | ,011 | ,001 | ,021 | ,000 | ,001 |
| COV2\_1 | <--> | ttDASS21ANKSIOZNOST | ,014 | ,001 | ,038 | ,000 | ,001 |
| T\_bracni\_status\_1 | <--> | ttDASS21ANKSIOZNOST | ,009 | ,000 | -,022 | ,000 | ,001 |
| ttDASS21DEPRESIJA | <--> | COV4\_1 | ,011 | ,001 | ,020 | ,000 | ,001 |
| COV3\_1 | <--> | T\_zaposlena\_1 | ,012 | ,001 | ,019 | -,001 | ,001 |
| T\_bracni\_status\_1 | <--> | COV4\_1 | ,010 | ,001 | -,023 | -,001 | ,001 |
| COV4\_1 | <--> | T\_zaposlena\_1 | ,010 | ,000 | -,020 | ,001 | ,001 |

##### Correlations: (Group number 1 - Default model)

| Parameter | | | SE | SE-SE | Mean | Bias | SE-Bias |
| --- | --- | --- | --- | --- | --- | --- | --- |
| ttDASS21ANKSIOZNOST | <--> | ttDASS21STRES | ,037 | ,002 | ,477 | -,004 | ,003 |
| ttDASS21DEPRESIJA | <--> | ttDASS21ANKSIOZNOST | ,053 | ,003 | ,499 | -,001 | ,004 |
| ttDASS21DEPRESIJA | <--> | ttDASS21STRES | ,055 | ,003 | ,549 | ,004 | ,004 |
| ttDASS21STRES | <--> | COV4\_1 | ,067 | ,003 | ,224 | -,008 | ,005 |
| T\_bracni\_status\_1 | <--> | T\_zaposlena\_1 | ,063 | ,003 | ,187 | ,004 | ,004 |
| COV2\_1 | <--> | ttDASS21STRES | ,057 | ,003 | ,108 | -,005 | ,004 |
| ttDASS21STRES | <--> | T\_zaposlena\_1 | ,058 | ,003 | -,207 | ,001 | ,004 |
| T\_bracni\_status\_1 | <--> | ttDASS21STRES | ,075 | ,004 | -,277 | ,005 | ,005 |
| COV3\_1 | <--> | COV4\_1 | ,062 | ,003 | -,086 | ,000 | ,004 |
| T\_bracni\_status\_1 | <--> | ttDASS21DEPRESIJA | ,061 | ,003 | -,183 | -,003 | ,004 |
| ttDASS21DEPRESIJA | <--> | T\_zaposlena\_1 | ,051 | ,003 | -,161 | -,008 | ,004 |
| COV2\_1 | <--> | ttDASS21DEPRESIJA | ,062 | ,003 | ,189 | -,001 | ,004 |
| ttDASS21ANKSIOZNOST | <--> | COV4\_1 | ,060 | ,003 | ,122 | ,000 | ,004 |
| COV2\_1 | <--> | ttDASS21ANKSIOZNOST | ,059 | ,003 | ,168 | ,000 | ,004 |
| T\_bracni\_status\_1 | <--> | ttDASS21ANKSIOZNOST | ,055 | ,003 | -,143 | -,001 | ,004 |
| ttDASS21DEPRESIJA | <--> | COV4\_1 | ,067 | ,003 | ,125 | ,001 | ,005 |
| COV3\_1 | <--> | T\_zaposlena\_1 | ,059 | ,003 | ,096 | -,005 | ,004 |
| T\_bracni\_status\_1 | <--> | COV4\_1 | ,087 | ,004 | -,210 | -,007 | ,006 |
| COV4\_1 | <--> | T\_zaposlena\_1 | ,062 | ,003 | -,128 | ,007 | ,004 |

##### Variances: (Group number 1 - Default model)

| Parameter | | | SE | SE-SE | Mean | Bias | SE-Bias |
| --- | --- | --- | --- | --- | --- | --- | --- |
| T\_bracni\_status\_1 |  |  | ,014 | ,001 | ,092 | ,000 | ,001 |
| HTA |  |  | ,014 | ,001 | ,124 | -,002 | ,001 |
| COV2\_1 |  |  | ,011 | ,001 | ,209 | ,000 | ,001 |
| COV3\_1 |  |  | ,012 | ,001 | ,208 | ,000 | ,001 |
| ttDASS21DEPRESIJA |  |  | ,010 | ,000 | ,215 | -,001 | ,001 |
| ttDASS21ANKSIOZNOST |  |  | ,003 | ,000 | ,246 | -,001 | ,000 |
| ttDASS21STRES |  |  | ,015 | ,001 | ,138 | -,001 | ,001 |
| COV4\_1 |  |  | ,016 | ,001 | ,123 | ,001 | ,001 |
| T\_zaposlena\_1 |  |  | ,013 | ,001 | ,193 | -,001 | ,001 |
| e1 |  |  | 12,889 | ,644 | 156,627 | -4,818 | ,911 |
| e2 |  |  | ,392 | ,020 | 3,844 | -,083 | ,028 |

##### Squared Multiple Correlations: (Group number 1 - Default model)

| Parameter | | | SE | SE-SE | Mean | Bias | SE-Bias |
| --- | --- | --- | --- | --- | --- | --- | --- |
| TOTALIP |  |  | ,044 | ,002 | ,272 | ,018 | ,003 |
| WHOQOL\_DOM2\_PSYCHOLOGICAL |  |  | ,050 | ,003 | ,400 | ,008 | ,004 |

##### Matrices (Group number 1 - Default model)

##### Total Effects - Standard Errors (Group number 1 - Default model)

|  | ttDASS21STRES | ttDASS21ANKSIOZNOST | ttDASS21DEPRESIJA | COV3\_1 | COV2\_1 | HTA | T\_bracni\_status\_1 | TOTALIP |
| --- | --- | --- | --- | --- | --- | --- | --- | --- |
| TOTALIP | ,000 | 1,731 | 1,767 | 1,780 | 1,859 | 2,510 | 2,685 | ,000 |
| WHOQOL\_DOM2\_PSYCHOLOGICAL | ,451 | ,251 | ,374 | ,074 | ,087 | ,097 | ,129 | ,010 |

##### Standardized Total Effects - Standard Errors (Group number 1 - Default model)

|  | ttDASS21STRES | ttDASS21ANKSIOZNOST | ttDASS21DEPRESIJA | COV3\_1 | COV2\_1 | HTA | T\_bracni\_status\_1 | TOTALIP |
| --- | --- | --- | --- | --- | --- | --- | --- | --- |
| TOTALIP | ,000 | ,057 | ,054 | ,056 | ,057 | ,060 | ,055 | ,000 |
| WHOQOL\_DOM2\_PSYCHOLOGICAL | ,062 | ,052 | ,066 | ,013 | ,015 | ,013 | ,015 | ,059 |

##### Direct Effects - Standard Errors (Group number 1 - Default model)

|  | ttDASS21STRES | ttDASS21ANKSIOZNOST | ttDASS21DEPRESIJA | COV3\_1 | COV2\_1 | HTA | T\_bracni\_status\_1 | TOTALIP |
| --- | --- | --- | --- | --- | --- | --- | --- | --- |
| TOTALIP | ,000 | 1,731 | 1,767 | 1,780 | 1,859 | 2,510 | 2,685 | ,000 |
| WHOQOL\_DOM2\_PSYCHOLOGICAL | ,451 | ,277 | ,386 | ,000 | ,000 | ,000 | ,000 | ,010 |

##### Standardized Direct Effects - Standard Errors (Group number 1 - Default model)

|  | ttDASS21STRES | ttDASS21ANKSIOZNOST | ttDASS21DEPRESIJA | COV3\_1 | COV2\_1 | HTA | T\_bracni\_status\_1 | TOTALIP |
| --- | --- | --- | --- | --- | --- | --- | --- | --- |
| TOTALIP | ,000 | ,057 | ,054 | ,056 | ,057 | ,060 | ,055 | ,000 |
| WHOQOL\_DOM2\_PSYCHOLOGICAL | ,062 | ,057 | ,069 | ,000 | ,000 | ,000 | ,000 | ,059 |

##### Indirect Effects - Standard Errors (Group number 1 - Default model)

|  | ttDASS21STRES | ttDASS21ANKSIOZNOST | ttDASS21DEPRESIJA | COV3\_1 | COV2\_1 | HTA | T\_bracni\_status\_1 | TOTALIP |
| --- | --- | --- | --- | --- | --- | --- | --- | --- |
| TOTALIP | ,000 | ,000 | ,000 | ,000 | ,000 | ,000 | ,000 | ,000 |
| WHOQOL\_DOM2\_PSYCHOLOGICAL | ,000 | ,075 | ,094 | ,074 | ,087 | ,097 | ,129 | ,000 |

##### Standardized Indirect Effects - Standard Errors (Group number 1 - Default model)

|  | ttDASS21STRES | ttDASS21ANKSIOZNOST | ttDASS21DEPRESIJA | COV3\_1 | COV2\_1 | HTA | T\_bracni\_status\_1 | TOTALIP |
| --- | --- | --- | --- | --- | --- | --- | --- | --- |
| TOTALIP | ,000 | ,000 | ,000 | ,000 | ,000 | ,000 | ,000 | ,000 |
| WHOQOL\_DOM2\_PSYCHOLOGICAL | ,000 | ,014 | ,017 | ,013 | ,015 | ,013 | ,015 | ,000 |

##### Bootstrap Confidence (Group number 1 - Default model)

##### Percentile method (Group number 1 - Default model)

##### 90% confidence intervals (percentile method)

##### Scalar Estimates (Group number 1 - Default model)

##### Regression Weights: (Group number 1 - Default model)

| Parameter | | | Estimate | Lower | Upper | P |
| --- | --- | --- | --- | --- | --- | --- |
| TOTALIP | <--- | T\_bracni\_status\_1 | -7,599 | -11,932 | -3,173 | ,010 |
| TOTALIP | <--- | HTA | 5,320 | 1,486 | 9,716 | ,030 |
| TOTALIP | <--- | COV2\_1 | 6,453 | 3,145 | 9,521 | ,010 |
| TOTALIP | <--- | COV3\_1 | -5,856 | -8,888 | -2,872 | ,010 |
| TOTALIP | <--- | ttDASS21DEPRESIJA | 5,837 | 2,874 | 8,672 | ,010 |
| TOTALIP | <--- | ttDASS21ANKSIOZNOST | 5,172 | 2,246 | 8,163 | ,010 |
| WHOQOL\_DOM2\_PSYCHOLOGICAL | <--- | ttDASS21DEPRESIJA | -,931 | -1,570 | -,285 | ,010 |
| WHOQOL\_DOM2\_PSYCHOLOGICAL | <--- | ttDASS21ANKSIOZNOST | -,993 | -1,457 | -,524 | ,010 |
| WHOQOL\_DOM2\_PSYCHOLOGICAL | <--- | ttDASS21STRES | -1,986 | -2,742 | -1,261 | ,010 |
| WHOQOL\_DOM2\_PSYCHOLOGICAL | <--- | TOTALIP | -,030 | -,046 | -,013 | ,014 |

##### Standardized Regression Weights: (Group number 1 - Default model)

| Parameter | | | Estimate | Lower | Upper | P |
| --- | --- | --- | --- | --- | --- | --- |
| TOTALIP | <--- | T\_bracni\_status\_1 | -,157 | -,239 | -,058 | ,010 |
| TOTALIP | <--- | HTA | ,128 | ,034 | ,223 | ,030 |
| TOTALIP | <--- | COV2\_1 | ,200 | ,104 | ,294 | ,010 |
| TOTALIP | <--- | COV3\_1 | -,181 | -,273 | -,088 | ,010 |
| TOTALIP | <--- | ttDASS21DEPRESIJA | ,184 | ,092 | ,277 | ,010 |
| TOTALIP | <--- | ttDASS21ANKSIOZNOST | ,175 | ,078 | ,273 | ,010 |
| WHOQOL\_DOM2\_PSYCHOLOGICAL | <--- | ttDASS21DEPRESIJA | -,170 | -,283 | -,053 | ,010 |
| WHOQOL\_DOM2\_PSYCHOLOGICAL | <--- | ttDASS21ANKSIOZNOST | -,194 | -,293 | -,102 | ,010 |
| WHOQOL\_DOM2\_PSYCHOLOGICAL | <--- | ttDASS21STRES | -,292 | -,397 | -,182 | ,010 |
| WHOQOL\_DOM2\_PSYCHOLOGICAL | <--- | TOTALIP | -,173 | -,266 | -,074 | ,014 |

##### Covariances: (Group number 1 - Default model)

| Parameter | | | Estimate | Lower | Upper | P |
| --- | --- | --- | --- | --- | --- | --- |
| ttDASS21ANKSIOZNOST | <--> | ttDASS21STRES | ,089 | ,070 | ,105 | ,010 |
| ttDASS21DEPRESIJA | <--> | ttDASS21ANKSIOZNOST | ,115 | ,094 | ,136 | ,010 |
| ttDASS21DEPRESIJA | <--> | ttDASS21STRES | ,095 | ,072 | ,115 | ,010 |
| ttDASS21STRES | <--> | COV4\_1 | ,030 | ,012 | ,045 | ,010 |
| T\_bracni\_status\_1 | <--> | T\_zaposlena\_1 | ,025 | ,010 | ,040 | ,010 |
| COV2\_1 | <--> | ttDASS21STRES | ,019 | ,004 | ,036 | ,059 |
| ttDASS21STRES | <--> | T\_zaposlena\_1 | -,034 | -,050 | -,018 | ,010 |
| T\_bracni\_status\_1 | <--> | ttDASS21STRES | -,032 | -,049 | -,016 | ,010 |
| COV3\_1 | <--> | COV4\_1 | -,014 | -,030 | ,002 | ,137 |
| T\_bracni\_status\_1 | <--> | ttDASS21DEPRESIJA | -,025 | -,043 | -,012 | ,014 |
| ttDASS21DEPRESIJA | <--> | T\_zaposlena\_1 | -,031 | -,051 | -,015 | ,011 |
| COV2\_1 | <--> | ttDASS21DEPRESIJA | ,040 | ,018 | ,063 | ,015 |
| ttDASS21ANKSIOZNOST | <--> | COV4\_1 | ,021 | ,004 | ,039 | ,035 |
| COV2\_1 | <--> | ttDASS21ANKSIOZNOST | ,038 | ,015 | ,061 | ,010 |
| T\_bracni\_status\_1 | <--> | ttDASS21ANKSIOZNOST | -,021 | -,036 | -,007 | ,025 |
| ttDASS21DEPRESIJA | <--> | COV4\_1 | ,020 | ,002 | ,039 | ,082 |
| COV3\_1 | <--> | T\_zaposlena\_1 | ,020 | -,001 | ,038 | ,129 |
| T\_bracni\_status\_1 | <--> | COV4\_1 | -,022 | -,041 | -,007 | ,015 |
| COV4\_1 | <--> | T\_zaposlena\_1 | -,021 | -,036 | -,004 | ,043 |

##### Correlations: (Group number 1 - Default model)

| Parameter | | | Estimate | Lower | Upper | P |
| --- | --- | --- | --- | --- | --- | --- |
| ttDASS21ANKSIOZNOST | <--> | ttDASS21STRES | ,481 | ,415 | ,536 | ,010 |
| ttDASS21DEPRESIJA | <--> | ttDASS21ANKSIOZNOST | ,500 | ,408 | ,579 | ,010 |
| ttDASS21DEPRESIJA | <--> | ttDASS21STRES | ,546 | ,456 | ,635 | ,010 |
| ttDASS21STRES | <--> | COV4\_1 | ,232 | ,102 | ,342 | ,010 |
| T\_bracni\_status\_1 | <--> | T\_zaposlena\_1 | ,183 | ,075 | ,287 | ,010 |
| COV2\_1 | <--> | ttDASS21STRES | ,114 | ,025 | ,214 | ,059 |
| ttDASS21STRES | <--> | T\_zaposlena\_1 | -,208 | -,304 | -,117 | ,010 |
| T\_bracni\_status\_1 | <--> | ttDASS21STRES | -,282 | -,387 | -,162 | ,010 |
| COV3\_1 | <--> | COV4\_1 | -,087 | -,199 | ,008 | ,137 |
| T\_bracni\_status\_1 | <--> | ttDASS21DEPRESIJA | -,180 | -,279 | -,088 | ,014 |
| ttDASS21DEPRESIJA | <--> | T\_zaposlena\_1 | -,152 | -,244 | -,076 | ,011 |
| COV2\_1 | <--> | ttDASS21DEPRESIJA | ,189 | ,086 | ,290 | ,015 |
| ttDASS21ANKSIOZNOST | <--> | COV4\_1 | ,123 | ,024 | ,225 | ,035 |
| COV2\_1 | <--> | ttDASS21ANKSIOZNOST | ,168 | ,066 | ,264 | ,010 |
| T\_bracni\_status\_1 | <--> | ttDASS21ANKSIOZNOST | -,142 | -,233 | -,047 | ,026 |
| ttDASS21DEPRESIJA | <--> | COV4\_1 | ,124 | ,011 | ,237 | ,082 |
| COV3\_1 | <--> | T\_zaposlena\_1 | ,101 | -,003 | ,190 | ,129 |
| T\_bracni\_status\_1 | <--> | COV4\_1 | -,203 | -,360 | -,072 | ,016 |
| COV4\_1 | <--> | T\_zaposlena\_1 | -,136 | -,241 | -,023 | ,043 |

##### Variances: (Group number 1 - Default model)

| Parameter | | | Estimate | Lower | Upper | P |
| --- | --- | --- | --- | --- | --- | --- |
| T\_bracni\_status\_1 |  |  | ,092 | ,070 | ,115 | ,010 |
| HTA |  |  | ,126 | ,098 | ,147 | ,010 |
| COV2\_1 |  |  | ,209 | ,190 | ,225 | ,010 |
| COV3\_1 |  |  | ,207 | ,185 | ,226 | ,010 |
| ttDASS21DEPRESIJA |  |  | ,216 | ,196 | ,229 | ,010 |
| ttDASS21ANKSIOZNOST |  |  | ,247 | ,241 | ,249 | ,010 |
| ttDASS21STRES |  |  | ,139 | ,113 | ,164 | ,010 |
| COV4\_1 |  |  | ,123 | ,099 | ,150 | ,010 |
| T\_zaposlena\_1 |  |  | ,194 | ,172 | ,212 | ,010 |
| e1 |  |  | 161,445 | 134,416 | 176,978 | ,010 |
| e2 |  |  | 3,926 | 3,219 | 4,520 | ,010 |

##### Squared Multiple Correlations: (Group number 1 - Default model)

| Parameter | | | Estimate | Lower | Upper | P |
| --- | --- | --- | --- | --- | --- | --- |
| TOTALIP |  |  | ,255 | ,190 | ,345 | ,010 |
| WHOQOL\_DOM2\_PSYCHOLOGICAL |  |  | ,391 | ,310 | ,481 | ,010 |

##### Matrices (Group number 1 - Default model)

##### Total Effects (Group number 1 - Default model)

##### Total Effects - Lower Bounds (PC) (Group number 1 - Default model)

|  | ttDASS21STRES | ttDASS21ANKSIOZNOST | ttDASS21DEPRESIJA | COV3\_1 | COV2\_1 | HTA | T\_bracni\_status\_1 | TOTALIP |
| --- | --- | --- | --- | --- | --- | --- | --- | --- |
| TOTALIP | ,000 | 2,246 | 2,874 | -8,888 | 3,145 | 1,486 | -11,932 | ,000 |
| WHOQOL\_DOM2\_PSYCHOLOGICAL | -2,742 | -1,575 | -1,709 | ,054 | -,357 | -,333 | ,045 | -,046 |

##### Total Effects - Upper Bounds (PC) (Group number 1 - Default model)

|  | ttDASS21STRES | ttDASS21ANKSIOZNOST | ttDASS21DEPRESIJA | COV3\_1 | COV2\_1 | HTA | T\_bracni\_status\_1 | TOTALIP |
| --- | --- | --- | --- | --- | --- | --- | --- | --- |
| TOTALIP | ,000 | 8,163 | 8,672 | -2,872 | 9,521 | 9,716 | -3,173 | ,000 |
| WHOQOL\_DOM2\_PSYCHOLOGICAL | -1,261 | -,755 | -,479 | ,310 | -,058 | -,027 | ,487 | -,013 |

##### Total Effects - Two Tailed Significance (PC) (Group number 1 - Default model)

|  | ttDASS21STRES | ttDASS21ANKSIOZNOST | ttDASS21DEPRESIJA | COV3\_1 | COV2\_1 | HTA | T\_bracni\_status\_1 | TOTALIP |
| --- | --- | --- | --- | --- | --- | --- | --- | --- |
| TOTALIP | ... | ,010 | ,010 | ,010 | ,010 | ,030 | ,010 | ... |
| WHOQOL\_DOM2\_PSYCHOLOGICAL | ,010 | ,010 | ,010 | ,015 | ,015 | ,040 | ,019 | ,014 |

##### Standardized Total Effects (Group number 1 - Default model)

##### Standardized Total Effects - Lower Bounds (PC) (Group number 1 - Default model)

|  | ttDASS21STRES | ttDASS21ANKSIOZNOST | ttDASS21DEPRESIJA | COV3\_1 | COV2\_1 | HTA | T\_bracni\_status\_1 | TOTALIP |
| --- | --- | --- | --- | --- | --- | --- | --- | --- |
| TOTALIP | ,000 | ,078 | ,092 | -,273 | ,104 | ,034 | -,239 | ,000 |
| WHOQOL\_DOM2\_PSYCHOLOGICAL | -,397 | -,309 | -,309 | ,010 | -,064 | -,045 | ,006 | -,266 |

##### Standardized Total Effects - Upper Bounds (PC) (Group number 1 - Default model)

|  | ttDASS21STRES | ttDASS21ANKSIOZNOST | ttDASS21DEPRESIJA | COV3\_1 | COV2\_1 | HTA | T\_bracni\_status\_1 | TOTALIP |
| --- | --- | --- | --- | --- | --- | --- | --- | --- |
| TOTALIP | ,000 | ,273 | ,277 | -,088 | ,294 | ,223 | -,058 | ,000 |
| WHOQOL\_DOM2\_PSYCHOLOGICAL | -,182 | -,146 | -,087 | ,054 | -,010 | -,004 | ,058 | -,074 |

##### Standardized Total Effects - Two Tailed Significance (PC) (Group number 1 - Default model)

|  | ttDASS21STRES | ttDASS21ANKSIOZNOST | ttDASS21DEPRESIJA | COV3\_1 | COV2\_1 | HTA | T\_bracni\_status\_1 | TOTALIP |
| --- | --- | --- | --- | --- | --- | --- | --- | --- |
| TOTALIP | ... | ,010 | ,010 | ,010 | ,010 | ,030 | ,010 | ... |
| WHOQOL\_DOM2\_PSYCHOLOGICAL | ,010 | ,010 | ,010 | ,014 | ,015 | ,040 | ,019 | ,014 |

##### Direct Effects (Group number 1 - Default model)

##### Direct Effects - Lower Bounds (PC) (Group number 1 - Default model)

|  | ttDASS21STRES | ttDASS21ANKSIOZNOST | ttDASS21DEPRESIJA | COV3\_1 | COV2\_1 | HTA | T\_bracni\_status\_1 | TOTALIP |
| --- | --- | --- | --- | --- | --- | --- | --- | --- |
| TOTALIP | ,000 | 2,246 | 2,874 | -8,888 | 3,145 | 1,486 | -11,932 | ,000 |
| WHOQOL\_DOM2\_PSYCHOLOGICAL | -2,742 | -1,457 | -1,570 | ,000 | ,000 | ,000 | ,000 | -,046 |

##### Direct Effects - Upper Bounds (PC) (Group number 1 - Default model)

|  | ttDASS21STRES | ttDASS21ANKSIOZNOST | ttDASS21DEPRESIJA | COV3\_1 | COV2\_1 | HTA | T\_bracni\_status\_1 | TOTALIP |
| --- | --- | --- | --- | --- | --- | --- | --- | --- |
| TOTALIP | ,000 | 8,163 | 8,672 | -2,872 | 9,521 | 9,716 | -3,173 | ,000 |
| WHOQOL\_DOM2\_PSYCHOLOGICAL | -1,261 | -,524 | -,285 | ,000 | ,000 | ,000 | ,000 | -,013 |

##### Direct Effects - Two Tailed Significance (PC) (Group number 1 - Default model)

|  | ttDASS21STRES | ttDASS21ANKSIOZNOST | ttDASS21DEPRESIJA | COV3\_1 | COV2\_1 | HTA | T\_bracni\_status\_1 | TOTALIP |
| --- | --- | --- | --- | --- | --- | --- | --- | --- |
| TOTALIP | ... | ,010 | ,010 | ,010 | ,010 | ,030 | ,010 | ... |
| WHOQOL\_DOM2\_PSYCHOLOGICAL | ,010 | ,010 | ,010 | ... | ... | ... | ... | ,014 |

##### Standardized Direct Effects (Group number 1 - Default model)

##### Standardized Direct Effects - Lower Bounds (PC) (Group number 1 - Default model)

|  | ttDASS21STRES | ttDASS21ANKSIOZNOST | ttDASS21DEPRESIJA | COV3\_1 | COV2\_1 | HTA | T\_bracni\_status\_1 | TOTALIP |
| --- | --- | --- | --- | --- | --- | --- | --- | --- |
| TOTALIP | ,000 | ,078 | ,092 | -,273 | ,104 | ,034 | -,239 | ,000 |
| WHOQOL\_DOM2\_PSYCHOLOGICAL | -,397 | -,293 | -,283 | ,000 | ,000 | ,000 | ,000 | -,266 |

##### Standardized Direct Effects - Upper Bounds (PC) (Group number 1 - Default model)

|  | ttDASS21STRES | ttDASS21ANKSIOZNOST | ttDASS21DEPRESIJA | COV3\_1 | COV2\_1 | HTA | T\_bracni\_status\_1 | TOTALIP |
| --- | --- | --- | --- | --- | --- | --- | --- | --- |
| TOTALIP | ,000 | ,273 | ,277 | -,088 | ,294 | ,223 | -,058 | ,000 |
| WHOQOL\_DOM2\_PSYCHOLOGICAL | -,182 | -,102 | -,053 | ,000 | ,000 | ,000 | ,000 | -,074 |

##### Standardized Direct Effects - Two Tailed Significance (PC) (Group number 1 - Default model)

|  | ttDASS21STRES | ttDASS21ANKSIOZNOST | ttDASS21DEPRESIJA | COV3\_1 | COV2\_1 | HTA | T\_bracni\_status\_1 | TOTALIP |
| --- | --- | --- | --- | --- | --- | --- | --- | --- |
| TOTALIP | ... | ,010 | ,010 | ,010 | ,010 | ,030 | ,010 | ... |
| WHOQOL\_DOM2\_PSYCHOLOGICAL | ,010 | ,010 | ,010 | ... | ... | ... | ... | ,014 |

##### Indirect Effects (Group number 1 - Default model)

##### Indirect Effects - Lower Bounds (PC) (Group number 1 - Default model)

|  | ttDASS21STRES | ttDASS21ANKSIOZNOST | ttDASS21DEPRESIJA | COV3\_1 | COV2\_1 | HTA | T\_bracni\_status\_1 | TOTALIP |
| --- | --- | --- | --- | --- | --- | --- | --- | --- |
| TOTALIP | ,000 | ,000 | ,000 | ,000 | ,000 | ,000 | ,000 | ,000 |
| WHOQOL\_DOM2\_PSYCHOLOGICAL | ,000 | -,290 | -,372 | ,054 | -,357 | -,333 | ,045 | ,000 |

##### Indirect Effects - Upper Bounds (PC) (Group number 1 - Default model)

|  | ttDASS21STRES | ttDASS21ANKSIOZNOST | ttDASS21DEPRESIJA | COV3\_1 | COV2\_1 | HTA | T\_bracni\_status\_1 | TOTALIP |
| --- | --- | --- | --- | --- | --- | --- | --- | --- |
| TOTALIP | ,000 | ,000 | ,000 | ,000 | ,000 | ,000 | ,000 | ,000 |
| WHOQOL\_DOM2\_PSYCHOLOGICAL | ,000 | -,042 | -,058 | ,310 | -,058 | -,027 | ,487 | ,000 |

##### Indirect Effects - Two Tailed Significance (PC) (Group number 1 - Default model)

|  | ttDASS21STRES | ttDASS21ANKSIOZNOST | ttDASS21DEPRESIJA | COV3\_1 | COV2\_1 | HTA | T\_bracni\_status\_1 | TOTALIP |
| --- | --- | --- | --- | --- | --- | --- | --- | --- |
| TOTALIP | ... | ... | ... | ... | ... | ... | ... | ... |
| WHOQOL\_DOM2\_PSYCHOLOGICAL | ... | ,017 | ,019 | ,015 | ,015 | ,040 | ,019 | ... |

##### Standardized Indirect Effects (Group number 1 - Default model)

##### Standardized Indirect Effects - Lower Bounds (PC) (Group number 1 - Default model)

|  | ttDASS21STRES | ttDASS21ANKSIOZNOST | ttDASS21DEPRESIJA | COV3\_1 | COV2\_1 | HTA | T\_bracni\_status\_1 | TOTALIP |
| --- | --- | --- | --- | --- | --- | --- | --- | --- |
| TOTALIP | ,000 | ,000 | ,000 | ,000 | ,000 | ,000 | ,000 | ,000 |
| WHOQOL\_DOM2\_PSYCHOLOGICAL | ,000 | -,055 | -,064 | ,010 | -,064 | -,045 | ,006 | ,000 |

##### Standardized Indirect Effects - Upper Bounds (PC) (Group number 1 - Default model)

|  | ttDASS21STRES | ttDASS21ANKSIOZNOST | ttDASS21DEPRESIJA | COV3\_1 | COV2\_1 | HTA | T\_bracni\_status\_1 | TOTALIP |
| --- | --- | --- | --- | --- | --- | --- | --- | --- |
| TOTALIP | ,000 | ,000 | ,000 | ,000 | ,000 | ,000 | ,000 | ,000 |
| WHOQOL\_DOM2\_PSYCHOLOGICAL | ,000 | -,008 | -,011 | ,054 | -,010 | -,004 | ,058 | ,000 |

##### Standardized Indirect Effects - Two Tailed Significance (PC) (Group number 1 - Default model)

|  | ttDASS21STRES | ttDASS21ANKSIOZNOST | ttDASS21DEPRESIJA | COV3\_1 | COV2\_1 | HTA | T\_bracni\_status\_1 | TOTALIP |
| --- | --- | --- | --- | --- | --- | --- | --- | --- |
| TOTALIP | ... | ... | ... | ... | ... | ... | ... | ... |
| WHOQOL\_DOM2\_PSYCHOLOGICAL | ... | ,017 | ,019 | ,014 | ,015 | ,040 | ,019 | ... |

##### Minimization History (Default model)

| Iteration |  | Negative eigenvalues | Condition # | Smallest eigenvalue | Diameter | F | NTries | Ratio |
| --- | --- | --- | --- | --- | --- | --- | --- | --- |
| 0 | e | 3 |  | -,106 | 9999,000 | 723,598 | 0 | 9999,000 |
| 1 | e | 0 | 8,020 |  | ,995 | 120,695 | 18 | ,893 |
| 2 | e | 0 | 12,251 |  | ,459 | 57,177 | 1 | ,954 |
| 3 | e | 0 | 18,720 |  | ,301 | 38,021 | 1 | 1,163 |
| 4 | e | 0 | 24,614 |  | ,143 | 36,148 | 1 | 1,088 |
| 5 | e | 0 | 25,402 |  | ,027 | 36,105 | 1 | 1,020 |
| 6 | e | 0 | 25,688 |  | ,001 | 36,105 | 1 | 1,001 |

##### Pairwise Parameter Comparisons (Default model)

##### Correlations of Estimates (Default model)

|  | par\_1 | par\_2 | par\_3 | par\_4 | par\_5 | par\_6 | par\_7 | par\_8 | par\_9 | par\_10 | par\_11 | par\_12 | par\_13 | par\_14 | par\_15 | par\_16 | par\_17 | par\_18 | par\_19 | par\_20 | par\_21 | par\_22 | par\_23 | par\_24 | par\_25 | par\_26 | par\_27 | par\_28 | par\_29 | par\_30 | par\_31 | par\_32 | par\_33 | par\_34 | par\_35 | par\_36 | par\_37 | par\_38 | par\_39 | par\_40 |
| --- | --- | --- | --- | --- | --- | --- | --- | --- | --- | --- | --- | --- | --- | --- | --- | --- | --- | --- | --- | --- | --- | --- | --- | --- | --- | --- | --- | --- | --- | --- | --- | --- | --- | --- | --- | --- | --- | --- | --- | --- |
| par\_1 | 1,000 |
| par\_2 | ,000 | 1,000 |
| par\_3 | -,041 | ,000 | 1,000 |
| par\_4 | ,000 | ,000 | ,000 | 1,000 |
| par\_5 | ,131 | ,000 | -,127 | ,000 | 1,000 |
| par\_6 | ,065 | ,000 | -,089 | ,000 | -,470 | 1,000 |
| par\_7 | ,000 | ,000 | ,000 | ,000 | ,000 | ,000 | 1,000 |
| par\_8 | ,000 | ,000 | ,000 | ,000 | ,000 | ,000 | -,281 | 1,000 |
| par\_9 | ,000 | ,000 | ,000 | ,000 | ,000 | ,000 | -,389 | -,277 | 1,000 |
| par\_10 | ,000 | ,000 | ,000 | ,000 | ,000 | ,000 | -,185 | -,172 | -,033 | 1,000 |
| par\_11 | ,000 | ,000 | ,000 | ,000 | ,000 | ,000 | ,000 | ,000 | ,000 | ,000 | 1,000 |
| par\_12 | ,000 | ,000 | ,000 | ,000 | ,000 | ,000 | ,000 | ,000 | ,000 | ,000 | ,623 | 1,000 |
| par\_13 | ,000 | ,000 | ,000 | ,000 | ,000 | ,000 | ,000 | ,000 | ,000 | ,000 | ,590 | ,582 | 1,000 |
| par\_14 | ,000 | ,000 | ,000 | ,000 | ,000 | ,000 | ,000 | ,000 | ,000 | ,000 | ,191 | ,097 | ,201 | 1,000 |
| par\_15 | ,000 | ,000 | ,000 | ,000 | ,000 | ,000 | ,000 | ,000 | ,000 | ,000 | ,000 | ,000 | ,050 | ,065 | 1,000 |
| par\_16 | ,000 | ,000 | ,000 | ,000 | ,000 | ,000 | ,000 | ,000 | ,000 | ,000 | ,190 | ,153 | ,206 | ,000 | ,000 | 1,000 |
| par\_17 | ,000 | ,000 | ,000 | ,000 | ,000 | ,000 | ,000 | ,000 | ,000 | ,000 | ,000 | ,000 | -,179 | -,147 | -,279 | ,000 | 1,000 |
| par\_18 | ,000 | ,000 | ,000 | ,000 | ,000 | ,000 | ,000 | ,000 | ,000 | ,000 | -,221 | -,123 | -,265 | -,246 | -,236 | ,000 | ,202 | 1,000 |
| par\_19 | ,000 | ,000 | ,000 | ,000 | ,000 | ,000 | ,000 | ,000 | ,000 | ,000 | ,000 | ,000 | ,000 | ,000 | ,000 | ,000 | ,000 | ,000 | 1,000 |
| par\_20 | ,000 | ,000 | ,000 | ,000 | ,000 | ,000 | ,000 | ,000 | ,000 | ,000 | -,171 | -,187 | -,312 | -,131 | -,171 | ,000 | ,099 | ,552 | ,000 | 1,000 |
| par\_21 | ,000 | ,000 | ,000 | ,000 | ,000 | ,000 | ,000 | ,000 | ,000 | ,000 | ,000 | ,000 | -,204 | -,059 | -,159 | ,000 | ,424 | ,088 | ,000 | ,177 | 1,000 |
| par\_22 | ,000 | ,000 | ,000 | ,000 | ,000 | ,000 | ,000 | ,000 | ,000 | ,000 | ,121 | ,226 | ,175 | ,000 | ,000 | ,520 | ,000 | ,000 | ,000 | ,000 | ,000 | 1,000 |
| par\_23 | ,000 | ,000 | ,000 | ,000 | ,000 | ,000 | ,000 | ,000 | ,000 | ,000 | ,240 | ,146 | ,131 | ,479 | ,000 | ,000 | ,000 | -,111 | ,000 | -,101 | ,000 | ,000 | 1,000 |
| par\_24 | ,000 | ,000 | ,000 | ,000 | ,000 | ,000 | ,000 | ,000 | ,000 | ,000 | ,168 | ,237 | ,120 | ,000 | ,000 | ,478 | ,000 | ,000 | ,000 | ,000 | ,000 | ,507 | ,000 | 1,000 |
| par\_25 | ,000 | ,000 | ,000 | ,000 | ,000 | ,000 | ,000 | ,000 | ,000 | ,000 | -,284 | -,198 | -,168 | -,114 | ,000 | ,000 | ,000 | ,479 | ,000 | ,487 | ,000 | ,000 | -,199 | ,000 | 1,000 |
| par\_26 | ,000 | ,000 | ,000 | ,000 | ,000 | ,000 | ,000 | ,000 | ,000 | ,000 | ,146 | ,151 | ,249 | ,544 | ,043 | ,000 | -,074 | -,137 | ,000 | -,214 | -,121 | ,000 | ,487 | ,000 | -,106 | 1,000 |
| par\_27 | ,000 | ,000 | ,000 | ,000 | ,000 | ,000 | ,000 | ,000 | ,000 | ,000 | ,000 | ,000 | ,000 | ,000 | ,000 | ,000 | ,000 | ,000 | -,086 | ,000 | ,000 | ,000 | ,000 | ,000 | ,000 | ,000 | 1,000 |
| par\_28 | ,000 | ,000 | ,000 | ,000 | ,000 | ,000 | ,000 | ,000 | ,000 | ,000 | -,054 | -,030 | -,061 | -,315 | -,156 | ,000 | ,065 | ,273 | ,000 | ,154 | ,029 | ,000 | -,161 | ,000 | ,145 | -,200 | ,000 | 1,000 |
| par\_29 | ,000 | ,000 | ,000 | ,000 | ,000 | ,000 | ,000 | ,000 | ,000 | ,000 | ,000 | ,000 | -,035 | -,222 | -,206 | ,000 | ,221 | ,068 | ,103 | ,040 | ,096 | ,000 | ,000 | ,000 | ,000 | -,160 | -,090 | ,199 | 1,000 |
| par\_30 | ,000 | ,000 | ,000 | ,000 | ,000 | ,000 | ,000 | ,000 | ,000 | ,000 | ,045 | ,028 | ,058 | ,077 | ,254 | ,000 | -,062 | -,385 | ,000 | -,252 | -,033 | ,000 | ,037 | ,000 | -,199 | ,049 | ,000 | -,281 | -,048 | 1,000 |
| par\_31 | ,000 | ,000 | ,000 | ,000 | ,000 | ,000 | ,000 | ,000 | ,000 | ,000 | ,000 | ,000 | ,000 | ,000 | ,000 | ,000 | ,000 | ,000 | ,000 | ,000 | ,000 | ,000 | ,000 | ,000 | ,000 | ,000 | ,000 | ,000 | ,000 | ,000 | 1,000 |
| par\_32 | ,000 | ,000 | ,000 | ,000 | ,000 | ,000 | ,000 | ,000 | ,000 | ,000 | ,025 | ,041 | ,027 | ,000 | ,000 | ,171 | ,000 | ,000 | ,000 | ,000 | ,000 | ,270 | ,000 | ,238 | ,000 | ,000 | ,000 | ,000 | ,000 | ,000 | ,000 | 1,000 |
| par\_33 | ,000 | ,000 | ,000 | ,000 | ,000 | ,000 | ,000 | ,000 | ,000 | ,000 | ,000 | ,000 | ,000 | ,000 | ,000 | ,000 | ,000 | ,000 | -,127 | ,000 | ,000 | ,000 | ,000 | ,000 | ,000 | ,000 | ,148 | ,000 | -,012 | ,000 | ,000 | ,000 | 1,000 |
| par\_34 | ,000 | ,000 | ,000 | ,000 | ,000 | ,000 | ,000 | ,000 | ,000 | ,000 | ,333 | ,626 | ,672 | ,082 | ,024 | ,133 | -,073 | -,119 | ,000 | -,237 | -,185 | ,257 | ,071 | ,127 | -,103 | ,164 | ,000 | -,027 | -,014 | ,029 | ,000 | ,036 | ,000 | 1,000 |
| par\_35 | ,000 | ,000 | ,000 | ,000 | ,000 | ,000 | ,000 | ,000 | ,000 | ,000 | ,623 | ,637 | ,300 | ,079 | ,000 | ,109 | ,000 | -,091 | ,000 | -,095 | ,000 | ,113 | ,172 | ,230 | -,200 | ,083 | ,000 | -,024 | ,000 | ,020 | ,000 | ,028 | ,000 | ,250 | 1,000 |
| par\_36 | ,000 | ,000 | ,000 | ,000 | ,000 | ,000 | ,000 | ,000 | ,000 | ,000 | ,602 | ,317 | ,669 | ,311 | ,064 | ,150 | -,254 | -,373 | ,000 | -,198 | -,103 | ,076 | ,138 | ,070 | -,165 | ,166 | ,000 | -,086 | -,052 | ,075 | ,000 | ,013 | ,000 | ,285 | ,232 | 1,000 |
| par\_37 | ,000 | ,000 | ,000 | ,000 | ,000 | ,000 | ,000 | ,000 | ,000 | ,000 | ,033 | ,016 | ,033 | ,320 | ,033 | ,000 | -,035 | -,062 | -,117 | -,033 | -,013 | ,000 | ,173 | ,000 | -,032 | ,175 | ,009 | -,279 | -,188 | ,040 | ,000 | ,000 | ,007 | ,013 | ,015 | ,051 | 1,000 |
| par\_38 | ,000 | ,000 | ,000 | ,000 | ,000 | ,000 | ,000 | ,000 | ,000 | ,000 | ,000 | ,000 | ,040 | ,037 | ,257 | ,000 | -,325 | -,052 | -,010 | -,040 | -,246 | ,000 | ,000 | ,000 | ,000 | ,028 | ,136 | -,032 | -,190 | ,034 | ,000 | ,000 | ,010 | ,023 | ,000 | ,044 | ,017 | 1,000 |
| par\_39 | ,000 | ,000 | ,000 | ,000 | ,000 | ,000 | ,000 | ,000 | ,000 | ,000 | ,000 | ,000 | ,000 | ,000 | ,000 | ,000 | ,000 | ,000 | ,000 | ,000 | ,000 | ,000 | ,000 | ,000 | ,000 | ,000 | ,000 | ,000 | ,000 | ,000 | ,000 | ,000 | ,000 | ,000 | ,000 | ,000 | ,000 | ,000 | 1,000 |
| par\_40 | ,000 | ,000 | ,000 | ,000 | ,000 | ,000 | ,000 | ,000 | ,000 | ,000 | ,000 | ,000 | ,000 | ,000 | ,000 | ,000 | ,000 | ,000 | ,000 | ,000 | ,000 | ,000 | ,000 | ,000 | ,000 | ,000 | ,000 | ,000 | ,000 | ,000 | ,000 | ,000 | ,000 | ,000 | ,000 | ,000 | ,000 | ,000 | ,000 | 1,000 |

##### Bootstrap (Default model)

##### Summary of Bootstrap Iterations (Default model)

##### (Default model)

| Iterations | Method 0 | Method 1 | Method 2 |
| --- | --- | --- | --- |
| 1 | 0 | 0 | 0 |
| 2 | 0 | 0 | 0 |
| 3 | 0 | 0 | 0 |
| 4 | 0 | 0 | 0 |
| 5 | 0 | 0 | 0 |
| 6 | 0 | 0 | 0 |
| 7 | 0 | 7 | 0 |
| 8 | 0 | 51 | 0 |
| 9 | 0 | 77 | 0 |
| 10 | 0 | 44 | 0 |
| 11 | 0 | 15 | 0 |
| 12 | 0 | 4 | 0 |
| 13 | 0 | 2 | 0 |
| 14 | 0 | 0 | 0 |
| 15 | 0 | 0 | 0 |
| 16 | 0 | 0 | 0 |
| 17 | 0 | 0 | 0 |
| 18 | 0 | 0 | 0 |
| 19 | 0 | 0 | 0 |
| Total | 0 | 200 | 0 |

0 bootstrap samples were unused because of a singular covariance matrix.

0 bootstrap samples were unused because a solution was not found.

200 usable bootstrap samples were obtained.

##### Bootstrap Distributions (Default model)

##### ML discrepancy (implied vs sample) (Default model)

|  |  |  |
| --- | --- | --- |
|  |  | |-------------------- |
|  | 27,724 | |\* |
|  | 34,749 | |\*\*\* |
|  | 41,775 | |\*\*\*\*\*\*\*\* |
|  | 48,800 | |\*\*\*\*\*\*\*\*\*\*\*\* |
|  | 55,826 | |\*\*\*\*\*\*\*\*\*\*\*\*\*\*\*\*\*\*\* |
|  | 62,851 | |\*\*\*\*\*\*\*\*\*\*\*\*\*\*\*\*\*\*\* |
|  | 69,877 | |\*\*\*\*\*\*\*\*\*\*\*\*\*\*\*\* |
| N = 200 | 76,902 | |\*\*\*\*\*\*\*\*\*\*\*\*\* |
| Mean = 63,961 | 83,928 | |\*\*\*\*\*\* |
| S. e. = 1,122 | 90,954 | |\*\*\* |
|  | 97,979 | |\*\* |
|  | 105,005 | |\* |
|  | 112,030 | |\* |
|  | 119,056 | |\* |
|  | 126,081 | |\* |
|  |  | |-------------------- |

##### ML discrepancy (implied vs pop) (Default model)

|  |  |  |
| --- | --- | --- |
|  |  | |-------------------- |
|  | 61,206 | |\* |
|  | 68,840 | |\*\*\*\*\*\* |
|  | 76,474 | |\*\*\*\*\*\*\*\* |
|  | 84,108 | |\*\*\*\*\*\*\*\*\*\*\* |
|  | 91,742 | |\*\*\*\*\*\*\*\*\*\*\*\*\*\*\*\*\*\*\* |
|  | 99,376 | |\*\*\*\*\*\*\*\*\*\*\*\*\*\*\*\*\*\*\* |
|  | 107,010 | |\*\*\*\*\*\*\*\*\*\*\*\*\*\*\* |
| N = 200 | 114,644 | |\*\*\*\*\*\*\*\*\* |
| Mean = 100,330 | 122,278 | |\*\*\*\*\* |
| S. e. = 1,412 | 129,912 | |\*\*\* |
|  | 137,545 | |\* |
|  | 145,179 | |\*\*\* |
|  | 152,813 | |\* |
|  | 160,447 | |\*\* |
|  | 168,081 | |\* |
|  |  | |-------------------- |

##### K-L overoptimism (unstabilized) (Default model)

|  |  |  |
| --- | --- | --- |
|  |  | |-------------------- |
|  | -113,191 | |\* |
|  | -73,676 | |\*\*\* |
|  | -34,161 | |\*\*\*\*\*\* |
|  | 5,355 | |\*\*\*\*\*\*\*\*\*\*\* |
|  | 44,870 | |\*\*\*\*\*\*\*\*\*\* |
|  | 84,385 | |\*\*\*\*\*\*\*\*\*\*\*\*\*\*\* |
|  | 123,900 | |\*\*\*\*\*\*\*\*\*\*\*\* |
| N = 200 | 163,415 | |\*\*\*\*\*\*\*\*\*\*\*\*\*\*\*\* |
| Mean = 126,586 | 202,930 | |\*\*\*\*\*\*\*\* |
| S. e. = 7,967 | 242,445 | |\*\*\*\*\*\*\* |
|  | 281,961 | |\*\*\*\*\* |
|  | 321,476 | |\*\*\*\*\* |
|  | 360,991 | |\* |
|  | 400,506 | |\*\*\* |
|  | 440,021 | |\* |
|  |  | |-------------------- |

##### K-L overoptimism (stabilized) (Default model)

|  |  |  |
| --- | --- | --- |
|  |  | |-------------------- |
|  | 36,217 | |\* |
|  | 50,937 | |\* |
|  | 65,657 | |\* |
|  | 80,377 | |\*\*\*\*\*\* |
|  | 95,097 | |\*\*\*\*\*\*\*\*\*\* |
|  | 109,817 | |\*\*\*\*\*\*\*\*\*\*\*\*\* |
|  | 124,537 | |\*\*\*\*\*\*\*\*\*\*\*\*\*\*\*\* |
| N = 200 | 139,257 | |\*\*\*\*\*\*\* |
| Mean = 123,879 | 153,977 | |\*\*\*\*\*\*\* |
| S. e. = 2,485 | 168,697 | |\*\*\* |
|  | 183,417 | |\* |
|  | 198,137 | |\*\* |
|  | 212,857 | |\*\* |
|  | 227,577 | |\* |
|  | 242,297 | |\* |
|  |  | |-------------------- |

##### Model Fit Summary

##### CMIN

| Model | NPAR | CMIN | DF | P | CMIN/DF |
| --- | --- | --- | --- | --- | --- |
| Default model | 40 | 36,105 | 26 | ,090 | 1,389 |
| Saturated model | 66 | ,000 | 0 |
| Independence model | 11 | 605,131 | 55 | ,000 | 11,002 |

##### RMR, GFI

| Model | RMR | GFI | AGFI | PGFI |
| --- | --- | --- | --- | --- |
| Default model | 1,214 | ,978 | ,944 | ,385 |
| Saturated model | ,000 | 1,000 |  |  |
| Independence model | 2,031 | ,616 | ,539 | ,513 |

##### Baseline Comparisons

| Model | NFI Delta1 | RFI rho1 | IFI Delta2 | TLI rho2 | CFI |
| --- | --- | --- | --- | --- | --- |
| Default model | ,940 | ,874 | ,983 | ,961 | ,982 |
| Saturated model | 1,000 |  | 1,000 |  | 1,000 |
| Independence model | ,000 | ,000 | ,000 | ,000 | ,000 |

##### Parsimony-Adjusted Measures

| Model | PRATIO | PNFI | PCFI |
| --- | --- | --- | --- |
| Default model | ,473 | ,445 | ,464 |
| Saturated model | ,000 | ,000 | ,000 |
| Independence model | 1,000 | ,000 | ,000 |

##### NCP

| Model | NCP | LO 90 | HI 90 |
| --- | --- | --- | --- |
| Default model | 10,105 | ,000 | 30,047 |
| Saturated model | ,000 | ,000 | ,000 |
| Independence model | 550,131 | 474,751 | 632,960 |

##### FMIN

| Model | FMIN | F0 | LO 90 | HI 90 |
| --- | --- | --- | --- | --- |
| Default model | ,125 | ,035 | ,000 | ,104 |
| Saturated model | ,000 | ,000 | ,000 | ,000 |
| Independence model | 2,094 | 1,904 | 1,643 | 2,190 |

##### RMSEA

| Model | RMSEA | LO 90 | HI 90 | PCLOSE |
| --- | --- | --- | --- | --- |
| Default model | ,037 | ,000 | ,063 | ,771 |
| Independence model | ,186 | ,173 | ,200 | ,000 |

##### AIC

| Model | AIC | BCC | BIC | CAIC |
| --- | --- | --- | --- | --- |
| Default model | 116,105 | 119,571 | 262,900 | 302,900 |
| Saturated model | 132,000 | 137,718 | 374,212 | 440,212 |
| Independence model | 627,131 | 628,084 | 667,500 | 678,500 |

##### ECVI

| Model | ECVI | LO 90 | HI 90 | MECVI |
| --- | --- | --- | --- | --- |
| Default model | ,402 | ,367 | ,471 | ,414 |
| Saturated model | ,457 | ,457 | ,457 | ,477 |
| Independence model | 2,170 | 1,909 | 2,457 | 2,173 |

##### HOELTER

| Model | HOELTER .05 | HOELTER .01 |
| --- | --- | --- |
| Default model | 312 | 366 |
| Independence model | 36 | 40 |

##### Execution time summary

|  |  |
| --- | --- |
| Minimization: | ,016 |
| Miscellaneous: | ,354 |
| Bootstrap: | ,183 |
| Total: | ,553 |
