# Supplementary material for: First Balkan Brief Illness Perception Questionnaire (IPQ-B) among high-risk pregnancies
Source: PLoS One. 2025 Oct 28;20(10):e0334844. doi: 10.1371/journal.pone.0334844 (PMC12561911; doi:10.1371/journal.pone.0334844)
Supplement: S4 File — (PDF) [file pone.0334844.s004.pdf]

Appendix A. The Brief Illness Perception Questionnaire

For the following questions, please circle the number that best corresponds to your views:

How much does your illness affect your life?

0	1	2	3	4	5	6	7	8	9	10
no affect at all										severely affects my life

How long do you think your illness will continue?

0	1	2	3	4	5	6	7	8	9	10
a very short time										forever

How much control do you feel you have over your illness?

0	1	2	3	4	5	6	7	8	9	10
absolutely no control										extreme amount of control

How much do you think your treatment can help your illness?

0	1	2	3	4	5	6	7	8	9	10
not at all										extremely helpful

How much do you experience symptoms from your illness?

0	1	2	3	4	5	6	7	8	9	10
no symptoms at all										many severe symptoms

How concerned are you about your illness?

0	1	2	3	4	5	6	7	8	9	10
not at all concerned										extremely concerned

How well do you feel you understand your illness?

0	1	2	3	4	5	6	7	8	9	10
don't understand at all										understand very clearly

How much does your illness affect you emotionally? (e.g. does it make you angry, scared, upset or depressed?)

0	1	2	3	4	5	6	7	8	9	10
not at all affected emotionally										extremely affected emotionally

Please list in rank-order the three most important factors that you believe caused your illness.

The most important causes for me:-

1. ______________________________
2. ______________________________
3. ______________________________

Permission to use the existing questionnaire

Dear Dr Macura

Thanks for your email.

On behalf of my co-authors, I am happy for you to use the B-IPQ in your research, and hope that the study goes well.

With best wishes,

John Weinman
Professor of Psychology as applied to Medicines
School of Cancer & Pharmaceutical Sciences
King's College London
